# Leaping the hurdles in developing regenerative treatments for the intervertebral disc from preclinical to clinical

**DOI:** 10.1002/jsp2.1027

**Published:** 2018-08-02

**Authors:** Abbey A. Thorpe, Frances C. Bach, Marianna A. Tryfonidou, Christine L. Le Maitre, Fackson Mwale, Ashish D. Diwan, Keita Ito

**Affiliations:** ^1^ Biomolecular Sciences Research Centre Sheffield Hallam University Sheffield UK; ^2^ Department of Clinical Sciences of Companion Animals, Faculty of Veterinary Medicine Utrecht University Utrecht the Netherlands; ^3^ Department of Surgery McGill University Montreal Canada; ^4^ Spine Service, Department of Orthopaedic Surgery St. George & Sutherland Clinical School, University of New South Wales Sydney Australia; ^5^ Orthopaedic Biomechanics Division, Department of Biomedical Engineering Eindhoven University of Technology Eindhoven the Netherlands; ^6^ Department of Orthopedics University Medical Centre Utrecht Utrecht the Netherlands

**Keywords:** intellectual property, intervertebral disc, low back pain, neck pain, regeneration, translation

## Abstract

Chronic back and neck pain is a prevalent disability, often caused by degeneration of the intervertebral disc. Because current treatments for this condition are less than satisfactory, a great deal of effort is being applied to develop new solutions, including regenerative strategies. However, the path from initial promising idea to clinical use is fraught with many hurdles to overcome. Many of the keys to success are not necessarily linked to science or innovation. Successful translation to clinic will also rely on planning and awareness of the hurdles. It will be essential to plan your entire path to clinic from the outset and to do this with a multidisciplinary team. Take advice early on regulatory aspects and focus on generating the proof required to satisfy regulatory approval. Scientific demonstration and societal benefits are important, but translation cannot occur without involving commercial parties, which are instrumental to support expensive clinical trials. This will only be possible when intellectual property can be protected sufficiently to support a business model. In this manner, commercial, societal, medical, and scientific partners can work together to ultimately improve patient health. Based on literature surveys and experiences of the co‐authors, this opinion paper presents this pathway, highlights the most prominent issues and hopefully will aid in your own translational endeavors.

## 1 DESIGNING FOR SUCCESS

When developing new regenerative therapies for the degenerated intervertebral disc (IVD), a major cause of back and neck pain, it is essential to involve multidisciplinary teams from concept, through translation from bench to bedside, and even clinical application. The involvement of end users, including clinicians and patient groups, in the early stage of development is important to ensure that developed therapies will be applicable in the clinic and address an area of unmet need. End user engagement is also often required when applying for ethical permission for use of human tissues and clinical trial applications.^1^ Furthermore, engagement with end users is often required during design of research phases for funding, especially within Europe. The (regulatory) pathway to clinic is also important to consider from the outset, attention to appropriate laboratory tests to demonstrate proof of concept and initial toxicity and animal testing. Early considerations of commercialization, patenting and regulatory approvals are necessary to reduce delays to clinical trials and pathways to the clinic. Throughout laboratory and animal testing, good laboratory practice (GLP) or similar is required to support findings, regulatory approvals and patent applications. This includes aspects of good record keeping to laboratory maintenance and quality control.^2^ The design of clinical trials, stratification of patients and outcome measure design are all important to ensure that the correct questions are addressed at the appropriate time.

This review aims to map out the important aspects to consider during the development of regenerative therapies for the IVD. Covering the developmental stages for a diverse range of regenerative approaches including: gene, cell, biological factors, and biomaterial approaches to repair and regenerate the IVD, as a potential treatment for chronic back and neck pain.

## 2 BENCH SIDE TESTING: IN VITRO AND EX VIVO TESTING

**Figure 1:**
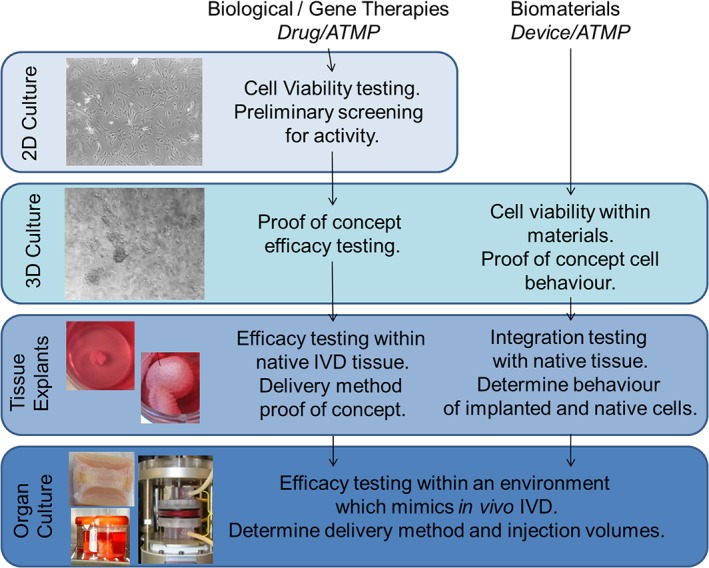
Recommended culture systems for developmental stages in regenerative therapy developments for the intervertebral disc (IVD). Regulatory classifications are shown in italics. ATMP, Advanced Therapeutic Medical Product. Images are representative images of culture systems: 2D culture: IVD cells in monolayer; 3D culture: IVD cells in alginate culture; tissue explants: explant culture systems; organ culture: examples of organ culture systems^3^, ^4^, ^5^, ^6^

The first stage in developing a new regenerative therapy for the IVD is testing in vitro, often in mammalian cell culture. This stage is used to perform initial proof of principle studies and fundamental cellular toxicology (viability) studies on relevant cell types. Important aspects to consider during the design of in vitro and ex vivo testing includes: species, sources of cells and tissues, and culture conditions. The specific question being addressed and stage within the developmental pathway will require different levels of complexity (Figure 1).

### 2.1 Species and cell/tissue source

**Figure 2:**
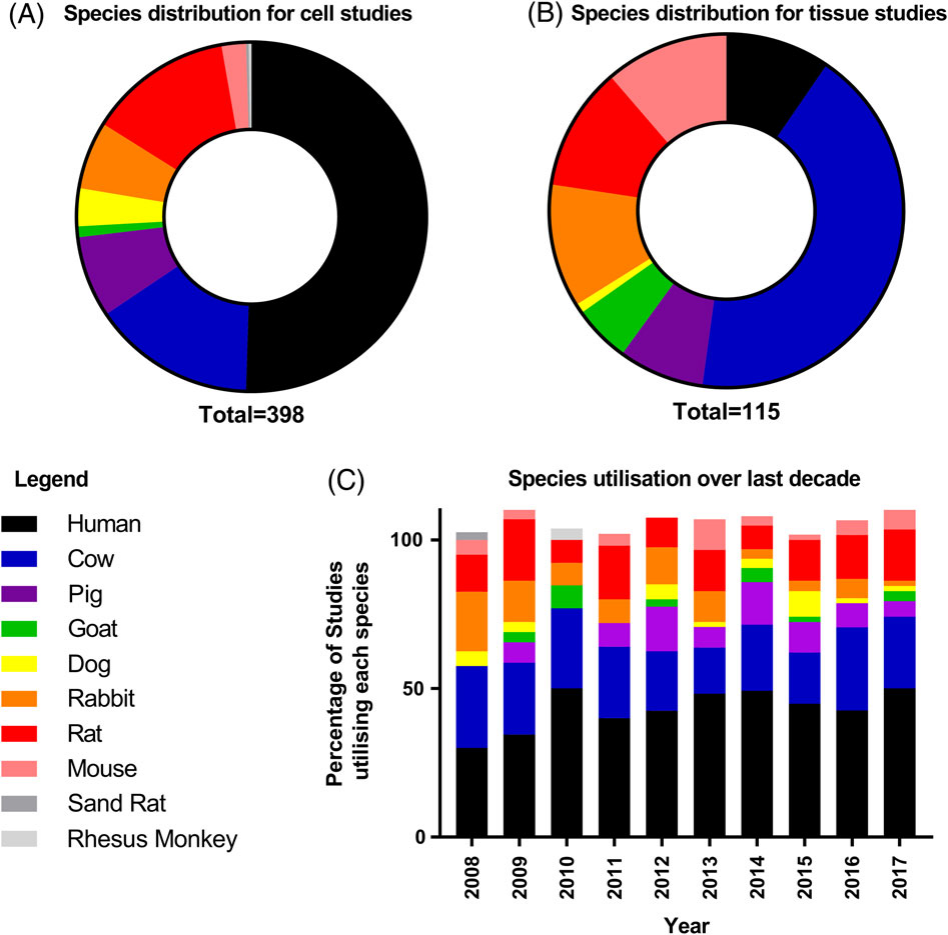
Utilization of cells and tissues for in vitro studies on intervertebral disc pathology and regeneration from 2008 to 2017. Results generated from a literature search for papers published over the last 10 years for “intervertebral disc culture.” (A) Species utilized for studies with isolated cells, (B) species utilized for tissue explant and organ cultures, (C) overall utilization in in vitro studies during the period of 2008‐2017

The choice of cell source is an important question. It is well known that species‐specific responses can be observed^7^, ^8^ and as such, caution must be applied when using nonhuman cells in initial proof of principle testing. These differences could arise from differential species‐dependent expression of proteins and receptors, cell types isolated from tissues (eg, notochordal cells), or the disease status of the tissues.^9^, ^10^, ^11^ In view of the challenges in accessing human cells, the majority of animal cells utilized in initial experimental studies are from normal young adult animals, which do not represent the cellular phenotype seen within the degenerated (human) IVD, which are senescent and exhibit a catabolic phenotype which has implications on regenerative therapies.^12^, ^13^, ^14^ Over the last 10 years, there has been increased research on human cell sources with approximately 50% of studies culturing IVD cells or cells for regeneration of the IVD sourced from human (Figure 2A,C). Accessibility to human tissues is more complex for tissue explant or organ culture studies with the majority of these utilizing animal tissues (Figure 2B), especially cow tails, due to their ease of access and similarity in cell type and structure to human discs.^15^ Similarities between animal and human disc tissues have been reviewed elsewhere.^15^ The utilization of animal tissues can be useful for initial tests and studies have attempted to mimic the degenerate disc within such systems (see below). However, no animal tissue represents the human situation directly. Therefore, human tissues should be utilized wherever possible, which also enables patient variation to be determined. Prior to translation to animal testing, it is also essential to confirm results in vitro within the species of choice for in vivo experimentation to ensure these cell types respond in the same way as human cells prior to animal model selection, as indicated recently, for example, by species‐specific responses to Link‐N.^8^

### 2.2 Culture systems

**Figure 3:**
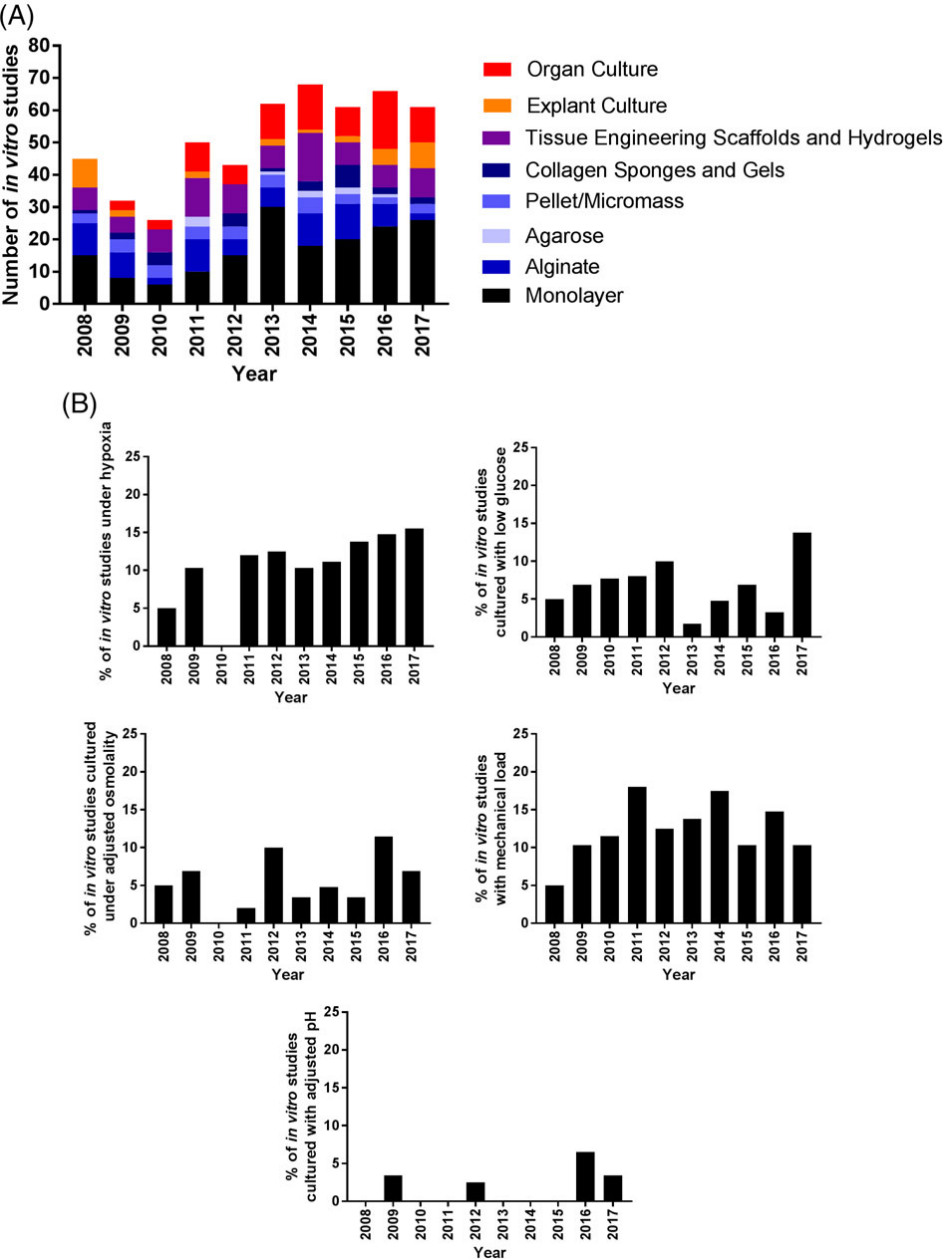
Culture conditions utilized from 2008 to 2017. Results generated from a literature search for papers published over the last 10 years for “intervertebral disc culture.” (A) Culture environment utilized (2D vs 3D vs tissue), (B) percentage of studies which modulated environmental conditions to mimic the intervertebral disc environment

Two‐dimensional (monolayer) culture of nucleus pulposus (NP) cells is well known to lead to changes in cellular phenotype, characterized by rapid de‐differentiation within the first passage, for example, loss of normal matrix synthesis (collagen type II and aggrecan) and gain of collagen type I.^14^, ^16^ Moreover, it acts as an oversimplification of the cellular environment, lacking cell‐extracellular matrix (ECM) interactions. Two‐dimensional (2D) culture systems for IVD studies are widely used (Figure 3A). While 2D culture has its use, particularly in initial cellular toxicology studies and preliminary proof of concept studies, it is difficult to translate results from 2D culture directly to the in vivo environment. Three‐dimensional culture systems such as pellet^17^ or alginate^14^ have been shown to restore the phenotype (with appropriate expression of IVD matrix molecules) of native NP cells and collagen scaffolds for annulus fibrosus (AF),^18^ thus, are a useful model to study effects of gene therapy and biological factors. However, these systems are often limited to single cell types and with the exception of newly deposited matrix do not recapitulate the complexity of cellular and extracellular matrix components and interactions thereof, which exist in vivo. The IVD regeneration field would benefit from defining a standard culture system such as alginate for NP and collagen for AF cells and its conditions (Table 1) that would be used worldwide and as such enable comparison of efficacy results. However, the defined “gold standard” will still need adjustments depending on the clinical questions addressed, as conditions in the IVD differ depending on age, spinal level, health state and underlying disease process.

**Table 1:** Recommended culture conditions to mimic the normal and degenerated intervertebral disc (IVD) environment

	Normal IVD	Degenerate IVD
Oxygen tension (%)^19^, ^20^	1‐5	1‐5
Glucose concentration (nM/mm^3^)^21^	0.94‐4	0.94‐4
Osmolality (mOsm/kg)^22^	400‐500	350
pH^23^, ^24^	7.0‐7.2	6.5‐7.1
Loaded environment	Dynamic load	Dynamic load
Catabolic factors^25^, ^26^		Cytokines (particularly IL‐1; 100 pg/mL), Ca^2+^ (2.5‐5 mM), or use of naturally degenerate cells/tissue within 3D culture, explants and organ cultures

Note that for proper mimicking the degenerative environment in 3D hydrogel culture low density of cells should be employed; for explant and organ culture diffusion of oxygen and glucose into the disc should be considered and thus higher culture concentrations may be required to result in these internal concentrations.

Until early in the 21st century, culture of IVD tissue explants, especially NP tissue explants, were hampered by tissue swelling, loss of extracellular matrix, cellular phenotype and viability. However, a number of culture systems are now available which can maintain tissue explants of animal and human tissue in culture for prolonged periods of time. These systems either constrain tissue volume,^3^, ^27^, ^28^ culture tissue in raised osmotic pressures,^29^ or under compressive loading,^30^ which prevents tissue swelling, maintains tissue architecture and cellular phenotype. These systems are particularly useful as human tissue obtained from surgery can be utilized as small pieces of intact tissue and can be maintained in culture. Such systems have been employed to excellent effect to study proof of principle studies on regenerative approaches including biological,^31^, ^32^ cellular^33^, ^34^, ^35^, and injectable hydrogel systems.^36^ These studies can provide useful initial data on local tissue responses, integration and delivery to tissues, which are essential in the pathway to clinic.

However, NP explants fail to model the interactions of different cell types within the disc and nutritional diffusion. Although this can be simulated to a certain extent by adjusting the nutrient supply in the media, this does not mimic the gradient seen in a whole IVD. Thus, a number of organ culture bioreactors have been developed which can maintain whole discs: mainly mouse, rabbit, sheep, cow, and goat discs. Recently, a long‐term IVD organ culture model that retains the vertebral bone system was developed.^6^ This model is useful for testing potential drugs on disc repair^37^ and is based on the bovine IVD. To study repair, IVDs are maintained in organ culture and degradation is induced by injection with trypsin. The whole organ culture system used for the bovine work is to some extent applicable to human IVDs, but in this case degeneration is not truly reflective of the human IVD. A number of systems have recently been developed which can maintain whole cadaveric human discs, which can allow investigations in naturally degenerate tissues.^38^, ^39^, ^40^ These systems have been reviewed in a number of excellent reviews.^41^, ^42^

### 2.3 Culture conditions

The native IVD in vivo is a hostile environment, characterized by low oxygen tension, low nutrition, high osmolality, low pH and exists under dynamic load^43^, ^44^ (Table 1). Yet the majority of in vitro studies are performed in nutrient‐rich culture media, most commonly Dulbecco's Modified Eagle Medium (DMEM) or DMEM/F12 consisting of high glucose concentrations, at neutral pH (7.4), low osmolality (~350 mOsm/kg), and under static culture conditions at 21% O_2_. The NP experiences mostly hydrostatic pressure, as high as 2.5 MPa, whereas the AF is under complex loading leading to direction‐dependent tensile, compression and shear stresses. The magnitude, duration and frequency of tissue loading, and deformation varies over a diurnal cycle.

Culture conditions are also essential to consider during expansion of cells for biobanking of IVD cells for regenerative approaches. Expansion conditions, including passage number, oxygen tension, supplements, and osmolality have been shown to influence the cell phenotype and as such influence the regenerative capacity and differentiation of mesenchymal stromal cells^45^ and NP cells.^46^ These conditions can be tuned, either to achieve optimal regenerative performance or to achieve an NP phenotype that resembles better the NP cells present within a degenerative niche.

During testing of regenerative approaches, systems should be tested within conditions which mimic the native IVD environment (Table 1). However, key factors preventing many researchers from modulating culture conditions are the comparison to previously published data and facilities that are available. Some studies are indeed starting to modulate these conditions within in vitro culture studies (Figure 3B). A key feature, however, which currently hampers in vitro culture modifications is accurate determination of actual levels seen in vivo.^47^ Furthermore, these conditions are known to change during degeneration, but levels will vary between patients and across regions within the IVD, and these measures are very difficult to determine in vivo and often depend on computer modeling to provide suggested concentrations.^44^ However, in order to gain a more educated understanding of how potential regenerative therapies will behave within the complex environment of the degenerated human IVD, in vitro and ex vivo culture systems must evolve to recapitulate the conditions seen within the degenerated IVD.

## 3 MODELING THE DEGENERATIVE NICHE IN VITRO

The degenerated IVD is a hostile environment for cells with further decrease in nutrients and pH and altered osmolarity compared to normal discs, which was recently reviewed by Sakai and Anderson.^43^ The degenerated niche also contains abundant catabolic cytokines, degradative enzymes, matrix fragments increased levels of free calcium (Ca^2+^),^6^ neurotrophic and angiogenic factors, which together could alter the behavior of any proposed regenerative therapy.^48^ For therapies which rely on the native cells of the IVD, these become senescent,^13^ alter phenotype, and undergo apoptosis, which results in an altered and/or reduced cell source available to respond to potential gene and biological treatments.^48^ While newly implanted cell sources may not survive and/or differentiate into the correct NP cell phenotype within the catabolic environment of the degenerate disc.^49^ Hence, it is important to assess any potential regenerative therapy within an environment which mimics the degenerated niche as much as possible (Table 1) prior to progression to clinical trials. ex vivo tissue explant and organ culture systems have in part begun to re‐create this niche with various degrees of success. Methods to mimic the changes observed in human IVD degeneration include enzymatic NP digestion,^50^ surgical methods to create AF injury,^51^ and overloading (by magnitude, duration, and frequency).^52^ However, these systems can only replicate some morpho‐histo‐pathological and cellular changes and it is unknown how closely they mimic/induce in vivo degenerative mechanisms. To date, the best ex vivo model systems available are those based on human degenerative IVD tissue/organs, but even these do not fully recapitulate the full in vivo environment as they are decoupled from systemic interactions, for example, immune, nervous, and endocrine systems.

Efforts are underway to develop realistic computational models for the human IVD, so called “virtual human IVD”, with the aim of diagnosing and understanding IVD degeneration.^53^, ^54^ As a step between simplified in vitro culture experiments and more sophisticated ex vivo culture employing tissue explants or even whole tissue organs, in silico modeling could provide an avenue to further identify essential environmental and cellular aspects that need to be considered in follow‐up studies. Although the field is still in its infancy,^55^ this may have the potential of performing in silico clinical trials and may help to optimize and guide the rational design of therapeutic interventions.^56^

## 4 PRECLINICAL ANIMAL MODELS

**Figure 4:**
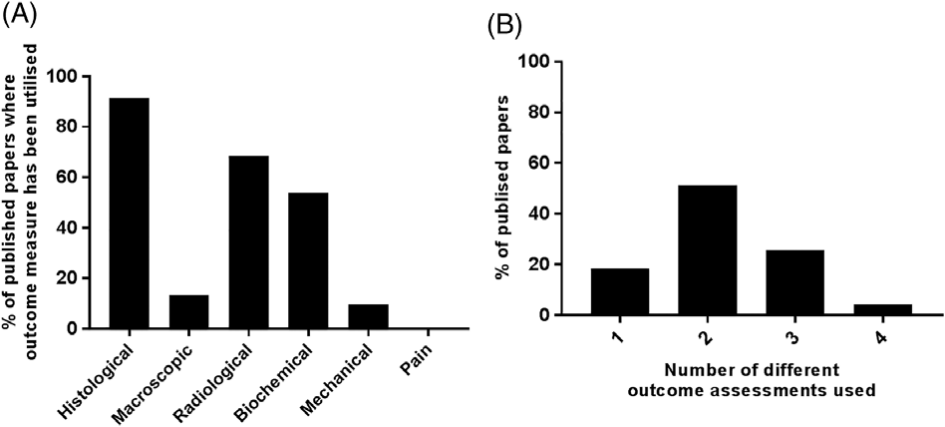
Results generated from a literature search for papers published over the last 20 years (1997‐2017) on regenerative treatments for the intervertebral disc in animal models. Hundred and twelve papers in total were reviewed and the outcome measures were separated into histological, macroscopic, radiological, biochemical, mechanical, and pain assessment. (A) Demonstrates the percentage (%) of these published papers that each of the different outcome measures were used in. (B) Demonstrates the number of different outcome measurements used within these publications

When promising (regenerative) treatment candidates have been established in vitro and/or in ex vivo tissue/organ cultures mimicking the degenerative disc niche, the next step would be to test these candidates in clinically relevant animal models for safety and efficacy prior to starting human clinical trials. To generate an overview of the types of efficacy outcome measures previously used, a literature search for papers published over the last 20 years on “regenerative treatments for the IVD in animal models” was performed. A total of 112 papers were reviewed and the outcome measures appeared to vary considerably between the different types of assessment (ie, histological, macroscopic, radiological, biochemical, mechanical, and pain assessment; Figure 4A,B). It is well known that IVD degeneration is a complex disease with cellular and biochemical matrix changes.^12^, ^57^, ^58^ Therefore, the assessment of histological and biochemical outcome measures is essential to fully evaluate the native cell response and matrix regeneration capacity of any treatment strategy. Despite this, histological, biochemical and/or radiological changes indicative of degeneration can be found in patients in the absence of pain, demonstrating that the two do not always correlate.^59^, ^60^, ^61^ Therefore, histological, biochemical and/or radiological improvements observed in animal models may not necessarily translate clinically into a reduction in disability and for this reason should not be used alone to indicate therapeutic success.

From a clinical point of view, the ultimate aim of any treatment developed for neck and back pain is to alleviate pain and restore the biomechanical function of the IVD. Interestingly, from the 112 papers reviewed, only 4% of these papers performed some kind of biomechanical assessment to determine the success of the therapy under investigation, and to the authors knowledge none of the papers reviewed had assessed pain as an outcome measure following administration of a regenerative therapy (Figure 4). This is likely due to the fact that standardized methods to assess biomechanical function and pain in animal models are less well defined. Furthermore, it is a common practice in translational studies to employ more than one levels within in each animal in order to reduce the number of animals needed in an experiment (3Rs principle: reduction, replacement, refinement). This limits the ability to assess pain properly. As such, once a promising treatment candidate has been encountered in studies with more than one spinal level injected with a different treatment, it would be recommended to perform an in vivo study on this treatment candidate injected at only one spinal level, enabling pain assessment.

Suitable methods to assess pain in animal models is still in its research infancy; for those that do exist, it is not clear whether these methods will relate to human neck and back pain, since the source of IVD‐related pain in humans is not always defined.^62^, ^63^ A number of preclinical small animal models that mimic specific aspects that contribute to low back pain (LBP) has been recently reviewed by Shi et al.^64^ Pain measurements in large animal models are primarily qualitative^65^ and deduced from objective gait analysis that does not allow for the exact (spinal) localization.^66^ Specifically in the case of dog patients with chronic back pain employed as a model for humans within the concept of “One Medicine,” owners can fill in questionnaires regarding pain assessment aspects and inherent impairment of mobility as would humans entering a clinical trial.^67^ Despite the difficulties, when evaluating the success of any potential therapy in animal models, it is recommended that some measure of biomechanical function and pain assessment, appropriate to the selected animal model, is performed. These outcome measures should be performed in combination with histological, biochemical and/or radiological outcome measures to evaluate the native IVD cell response, including production of catabolic/inflammatory factors, repair of matrix components and restoration of disc height. All outcomes should ideally be determined blindly and objectively, for example, by using quantitative scoring systems. This will improve knowledge concerning the efficacy of the therapeutic and may improve the translation of clinical findings within animal models to those found in humans. In this way, the chance of failure of the treatment candidate in human clinical trails would be reduced and translational success would be improved.

Commonly used experimental animal models for IVD degeneration include mice, rats, rabbits, dogs, sheep, goats,^15^ and more recently, alpacas have been employed.^68^, ^69^ Each animal model has distinct advantages and disadvantages, and therefore the choice of the animal model depends on the research question posed. It is important to note that no animal model can reproduce the exact nutritional status, biology, anatomy, and biomechanics of the human spine. Animal models differ considerably; there are even pronounced differences between animal breeds. It is evident that the difference in clinical representation of IVD‐related disease may strongly be related to the genetic background of the breeds (reviewed for dogs^70^). Similarly, also in humans, genetics play an important role as there have been “risk‐genes” identified in this sense. Although several predisposing genes have been reported (eg, aggrecan, collagen type I and XI, matrix metalloproteinase‐2, ‐3 and ‐9, cartilage intermediate layer protein, Interleukin‐1 and ‐6), only the association of vitamin D receptor (*VDR*) and collagen type IX (*COL9A2*) with IVD disease has been verified in different ethnic populations.^71^, ^72^ To our knowledge, no research has yet been performed on the IVD of VDR null or vitamin D‐deficient animals. Collagen type IX deficient mice, show early developmental, structural, and biomechanical alterations in their vertebral bodies and IVDs, causing severe degenerative changes in the aging spine.^73^ Most identified genes associated with LBP due to IVD degeneration code for proteins affecting ECM integrity, responsible for mechanical properties of the IVD. Thus far, animal studies on the genetics of IVD disease use mice.^74^, ^75^, ^76^ Although far less well researched, also in larger animal models employed for in vivo studies, genetics can play an important role.^77^ Therefore, researchers need to consider this when choosing a suitable animal model.

Differences in IVDs between human and animal species include anatomical (size, shape), biomechanical, biochemical, aging/degeneration, nutritional, cellular, and loading variations as reviewed by Alini et al.^15^ The difference in IVD size affects the type and number of readout parameters that can be measured: small IVDs cannot be used to evaluate multiple parameters and assay detection limits coincide with small samples. Also, limitations in relevant volume of therapeutics relative to tissue constructs are encountered.^78^ Although for large animal models this is not a specific issue, safe injection volumes and pressure should be determined to avoid injection‐induced accelerated degeneration.^79^ In this respect, IVD organ cultures are useful for injection volume and extrusion testing before animal models are employed. By using this approach, previous studies have demonstrated that no adverse effects were observed due to the intradiscal injections themselves, indicating that small volumes can be safely injected (rat: 1‐8 μL,^80^, ^81^, ^82^ rabbit: 10‐20 μL,^83^, ^84^, ^85^, ^86^, ^87^, ^88^ dog: 40‐50 μL,^67^, ^89^, ^90^ sheep: 0.2‐1.0 mL^91^, ^92^). We recommend injecting these IVD treatments only through the AF. Recent work has shown that the transpedicular approach, proposed as alternative delivery route for IVD regeneration,^93^ induced severe damage to the end plates and may lead to neurological impairment and leakage of injected material.^94^

Besides size, the second difference between animal models is that in most animals, degeneration needs to be induced, since this is not a spontaneously occurring phenomenon. Exceptions are the sand rat^95^, ^96^ and the dog,^70^ in which IVD degeneration occurs spontaneously with aging and is, at least in the dog, a clinical entity.^97^ In small animals, IVD degeneration can be induced by genetic modification,^74^, ^76^, ^98^ partial NP removal,^99^ IVD puncture,^100^, ^101^, ^102^ compression^103^, ^104^ (eg, tail‐looping^105^) and even whole body vibration.^106^ In large animal models, IVD degeneration can be induced by creating annular defects,^88^, ^107^, ^108^ partial NP removal^91^, ^109^, ^110^, and enzymatic NP digestion.^111^ A recently proposed option to induce NP damage is the use of laser technology,^111^ which induced more progressive and less pronounced IVD degeneration than enzymatic NP digestion. Researchers should thoroughly consider which IVD degeneration method to use, dependent on the research question(s), outcome parameters. and treatment candidate.

The third difference between species is the main cell type in the NP. Humans lose their notochordal cells (NCs) during childhood,^112^ whereas aging mice,^113^ (sand) rats,^96^, ^114^ rabbits,^115^ and nonchondrodystrophic (NCD) dogs^70^ typically still have NCs in their NP. In contrast, chondrodystrophic (CD) dogs,^70^ sheep,^116^ and goats^117^ lose their NCs early in life and therefore more closely resemble humans in this respect. Noteworthy, since in several animal species the main NP cell type changes with aging, the age of the experimental animals should be chosen cautiously.

In addition to interspecies differences, all common animal models are quadrupeds. The biomechanical forces exerted on the human IVD are often thought to be uniquely determined by the predominantly upright stature and bipedal locomotion of humans, and are therefore thought to be different to those found within in quadrupedal animal models. However, because of its segmental unstable nature, all spines rely on considerable tensile forces in intersegmental muscles (active) and ligaments (passive) to generate compressive loading on the anterior spinal column for sufficient stabilization. In quadrupeds, these anterior column compressive forces may in fact be higher than those in humans as demonstrated by the higher degree of longitudinal vertebral trabecular alignment and denser trabecular bone.^15^, ^118^ Of course, in addition to compressive loads, spinal segments must rotate in three degrees‐of‐freedom, and it is understandable to imagine that humans may require different motions from their spine than quadrupeds. However, when comparing the passive resistance to bending, Wilke et al found that the range‐of‐motion of sheep spinal segments for all load directions was qualitatively similar to that of humans.^119^ Nevertheless, when (regenerative) treatment candidates are tested in animal models in vivo, biomechanical testing in comparison to proper controls is recommended.

Altogether, it is extremely important to choose an appropriate animal model to test a (regenerative) treatment. The authors give recommendations in Table 2. Although small animals do not adequately represent humans considering the fact that IVD degeneration needs to be induced and the presence of NCs, they can be valuable for answering developmental questions, for example, by using genetic modification. Notably, a few ongoing clinical trials received an investigational new drug (IND) only based on small animal models. It remains to be determined whether this development in the regulatory scene is in the benefit of the patient. Two examples supporting this concept are recombinant human (rh) BMP7 and GDF5. Both were approved for intradiscal application based on small animal models. While a clinical trial Phase I/II was initiated for rhBMP7, it was never completed and results remain elusive. Furthermore, several clinical trials explored the efficacy of rhGDF‐5 and the results were inconclusive. Receiving an IND‐based on small animal models may make product development more affordable and shorten the time to market, but small animal models may insufficiently predict efficacy in man.

**Table 2:** Recommendations for the use of in vivo animal models

	Mouse	Rat	Rabbit	Sheep	Goat	Alpaca	CD dog	NCD dog
Cell type (CLC) in NP	−	−	−	++	++	ND	++	−
IVD size	−	−	−	+	+	+	−	+
Spontaneous IVD degeneration	−	−	−	−	−	−	++*	++*
Useful for fundamental/safety studies	++	++	++	−	−	−	−	−
Useful for translational/efficacy studies	(−)	(−)	(−)	+	+	+	++	++
Expenses for animal experiments	++	++	++	+	+	+	−	−

Abbreviations: CD, chondrodystrophic; CLC, chondrocyte‐like NP cell; NP, nucleus pulposus; IVD, intervertebral disc; NCD, nonchondrodystrophic.

++: Best suitable animal model for this specific purpose. +: Suitable animal model for this specific purpose. −: Less suitable animal model for this specific purpose. (−): Although the authors consider these species less suitable for this purpose, recent clinical trials (efficacy studies) did not require large animal studies. ND: not determined.

*: CD dog breeds typically develop IVD disease at relatively young age. NCD dog breeds can also develop IVD disease, but at an older age, mostly due to trauma or “wear and tear”. In the other species, IVD degeneration needs to be induced artificially.

When combining IVD size and NP cell type, sheep and goats more closely resemble the human situation, but IVD degeneration also needs to be induced. The question remains how adequately animal models with induced IVD degeneration represent the cellular changes which occur during natural degeneration in humans. Therefore, NCD or CD dogs may more accurately resemble human IVD degeneration where degeneration is spontaneous.^120^ Over the past few years, both human and veterinary medicine have recognized the importance of the “One Medicine” concept: bringing together human and animal health for new medical solutions, advantageous for humans as well as animals. An important issue of translating treatment strategies into preclinical animal models is the ethics of placebo treatment.^121^ Evidence‐based placebo treatment increases the scientific validity, but can in the case of an intradiscal sham injection pose risks and/or can lead to reluctance by the owners of the animals. Offering the option to provide the treatment to the patients that had previously received the placebo may increase the number of study participants.

A downside of using large animal models are the ethics and high costs (purchase and housing, multiple costly outcome parameters). Altogether, this often leads to the use of a minimal number of large animals included, impairing the power of the study. Furthermore, an issue concerning all species is the absence of histological scoring systems. To our knowledge, this has only been developed for mice^122^ and dogs.^123^ In terms of imaging, large animal models have rather similar possibilities as humans with LBP: radiography, fluoroscopy, discography, computer tomography (CT), and magnetic resonance imaging (MRI).^67^, ^90^, ^124^, ^125^ However, there are some drawbacks. For instance, quantitative MRI (eg, T1rho and T2 mapping) has been validated for human IVD degeneration,^126^, ^127^ but not for animals. Therefore, this needs to be validated for other species, as well as how the spinal phenotypes present in animal models relate to human pathology to improve translation. Another major concern regarding MRI analysis is that there is a need for validation of regenerative process, since quantitative MRI has specifically been validated for IVD degeneration, but not for regeneration, which does not necessarily follow an identical reverse process. To this end, the recently identified correlation between IVD degeneration, modic changes and back pain^128^, ^129^ indicates that in animal models too these entities need to be explored and properly characterized to fully cover the whole spectrum of spine pathology related to IVD degeneration. Lastly, long‐term animal studies are lacking, but must be performed to demonstrate long‐term safety and efficacy in clinically relevant animal models and detect pathological features that only develop after a long time period, such as tumorgenicity, before treatments are translated to human clinical trials. Regardless the approach, even if efficacy is demonstrated in a large animal model, it does not necessarily guarantee efficacy in the human patient. Considering the most recent developments regarding regulation and ethics concerning animal modeling, further developments in the preclinical track need to focus on implementation of the 3Rs principles, where replacement and considerable reduction of animal experiments needs to be achieved with sophisticated alternatives employing bioreactor technology mimicking the biology and biomechanics of the degenerative disc niche.

## 5 REGULATION

Regulatory pathways for the Food and Drug Administration (FDA), and Medicines and Healthcare products Regulatory Agency approval, will depend on the therapeutic under investigation and whether it is defined as a drug, biologic or device. When considering the well‐defined regulatory pathways for drugs, it is estimated that the average length of time from discovery to clinical application is approximately 12 to 15 years with an estimated cost of $800 million.^130^ In contrast, the regulatory pathways for biological therapeutic approaches are often more complex and time consuming. The FDA have established that biological drugs include blood‐derived products, vaccines, in vivo diagnostic allogenic products, immunoglobulin products, protein products and products containing cells or microorganisms.^131^ Given the unique nature of biological therapeutics, the preclinical tests performed to evaluate the safety, purity, potency and efficacy of the therapeutic will often be specific to the biological therapeutic under investigation. It is therefore recommended that researchers have contact with their local regulatory authorities early on in the preclinical experimental design process to ensure that the necessary experiments are being performed in line with the requirements for an IND application and premarket approval (biologics license application). Consideration, early on during preclinical investigations, should also be given to the manufacturing processes of the therapeutic. In comparison to well‐characterized synthetic small molecule drugs, regulatory authorities will often require additional clinical studies to demonstrate the identity, safety, purity, potency, and efficacy of the biologic following manufacturing processes.^132^

Currently, there is an increasing research interest for the use of implantable biomaterial scaffolds to replace tissues of the IVD as a treatment strategy for LBP.^133^, ^134^ Where the biomaterial scaffold is delivered without cells or biological factors it will likely be classified as an implantable medical device, which are typically subject to the regulatory requirements of class III medical devices (90/385/EEC).^135^ Again, it is essential that regulatory considerations are thought of early on, even while the initial in vitro investigations are being performed; this is because certain long‐term surveillance studies may be required for regulatory approval, for example, long‐term degradation and materials characterization studies in accordance with the ISO10993 standards.^136^ Where cells are either incorporated within biomaterials scaffolds or used individually for regenerative purposes, the therapy will likely be classified as an Advanced Therapy Medicinal Product.^137^ However, the classification of systems is different within each regulatory authority and is beyond the scope of this review to advise specific regulatory guidelines. Investigators are encouraged to contact their local regulatory bodies for advice as early as possible in the developmental pipeline, to enable appropriate investigations to be incorporated into development.

## 6 CLINICAL TRIAL DESIGN

**Figure 5:**
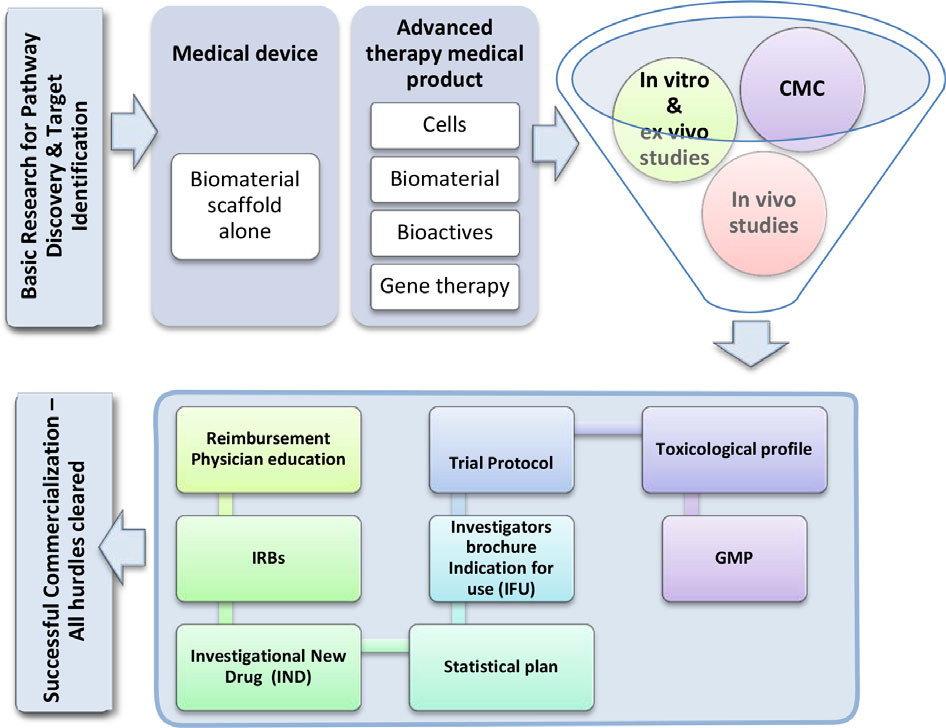
A road map of the pathway to clinical success of a potential intradiscal therapeutic agent. While each stage has hurdles of its own, comfort with acronyms and language around various steps and documentations needed is a good first step in resolving those hurdles. All activities may cumulatively take anywhere between 12 to 15 years. CMC, chemical and manufacturing control; GMP, good manufacturing practices; IND, investigational new drug; IFU, indication for use; IRB, institutional review board or ethics committees

Translating a potential product with great preclinical data to clinical reality, that is, from the bench to bedside, requires numerous steps. For a therapeutic agent that will be injected into the IVD under image guidance as a single dose, the pathway will be that for a new drug application, biological agent, medical device, or advanced therapy medical product. While the regulatory nuances can differ from one regime to another, some principles remain the same. Here, we describe numerous steps, documents, principles, and three‐lettered acronyms involved in completing the clinical translational work for regenerative therapies for the IVD (Figure 5). On identification of a suitable target, completion of proof of concept work, assuring a high quality Chemical and Manufacturing Control when needed, confirmation of preclinical toxicological work on the final product that will be used in clinical trials, with or without the requirement that the final product is made using Good Manufacturing Practices. A team with experience in early commercialization or clinical translation must be involved.

An Investigator Brochure is the first step. This document summarizes the history of the product development, characterization of the active pharmacological/biological ingredient, medical device or ATMP, mechanism of actions, all preclinical work and toxicological profiles (Table 3; list of toxicological work) and any functional pain studies performed. An indication for use (IFU) has to be stated clearly. It is important that all preclinical and proof of concept work is consistent and appropriate with this IFU.

**Table 3:** Toxicological and analytical work that may be required for investigational new drug (IND) application

Type of study	Model
Pharmacokinetics	
Intramuscular pharmacokinetics	Rat
Six‐month single dose safety study	Rat
Toxicology	
Pyrogen test	Rabbit
CNS safety profile	Rodents
Blood fibrinogen consumption test, platelet activation, complement activation test, hemolytic activity test	Human blood in vitro
Cardiovascular and pulmonary safety	Rodents
Intramuscular bone or tissue induction	Rodents
Effects on cell phenotype, metabolic activity, binding/affinity studies	In vitro depending on active ingredient (described above in preclinical studies)
Bioanalytical	
Dosing solution/delivery agent method development and validation	In vitro
Plasma assay development and validation	In vitro

The principles of understanding the pharmacodynamics and pharmacokinetics along with toxicological profile of the agent while being able to quantify the drug, its metabolite have to be demonstrated for other advanced therapies (including cell therapies), the toxicological and analytical work required is derived from the principles for drugs as listed.

The IFU becomes the basis of developing a Clinical Trial Protocol (CTP). This activity requires the input of clinicians who understand the Good Clinical Practice (GCP) for Trials and the Helsinki declaration. Trials are to be conducted on sites that are GCP compliant, as determined by the clinical trial sponsor from the initiation to completion of the study. The key elements of the CTP are: clear identification of the inclusion and exclusion criteria, clearly defined outcome measures and validation of the tools for clinical outcomes, a time table for what will be measured when, establishment of a Data Safety & Monitoring Board that can stop a trial in the event of a Serious Adverse Event, and a detailed Subject Information Sheet/Document. For patients undergoing a LBP study, each clinical trial protocol will be different, however, the minimum expectation for outcome tools will include a score for back pain, a disability measuring tool and a quality of life instrument (Table 4). Patient Reported Outcome Measures while generally accepted, are being questioned now in favor of subjectively quantifiable measures with the advent of wearable devices and potentially using biomarkers for disc degeneration.^138^

**Table 4:** Minimum outcome measures for a low back pain study

Minimum outcome measures	Example of scoring system/measurement
Pain	VAS, NRS
Disability	ODI, Roland Morris
Quality of life	SF36, EQ5
Radiological	DHI, MRI scans (if possible T1rho mapping)

Abbreviations: DHI, disc height index; EQ5, European quality (of life) 5 questions; MRI, magnetic resonance imaging; NRS, numeric rating scale; ODI, Oswestry disability index; SF36, 36‐item short form health survey; VAS, visual analog scale.

A clinical trial protocol has to consist of subjective (patient reported) and objective (investigator determined) outcome tools.

Radiological outcomes will be expected too, where the minimum will be a disc height measurement on a standing lateral X‐ray in neutral position. While (quantitative) MRI provides information on the degenerative stage of the IVD at the initiation of the study, the role of clinical MRI as outcome measures are uncertain and may not serve practical utility during a clinical trial. However, including MRI as a secondary read out parameter will assist follow up of the degenerative state of the treated disc and demonstrate the development or lack of additional pathologies, for example, modic changes. The role of endplate changes cannot be discounted but there is lack of consensus among researchers and clinicians as to their importance or predictive role in LBP. T1rho MRI mapping has been recently proposed as a marker for painful discs.^139^ However, lack of extensive clinical use and inadequate extensive validation of this imaging modality requires more work. Secondary outcome measures may include use of supplementary therapy and ability to work. Adverse events (AEs) are monitored throughout the trial and serious adverse events would include death, paralysis, infection and un‐remittent exacerbation of pain.

**Figure 6:**
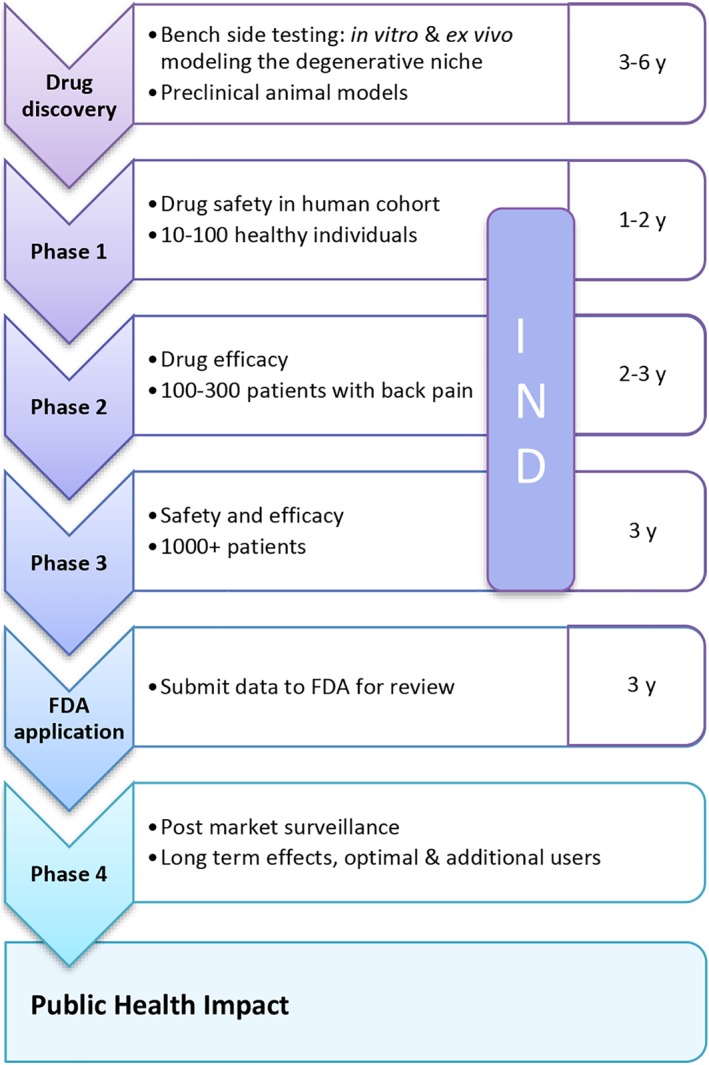
Drug approval process from bench to bedside. Phase I may not be needed for intradiscal therapies. Direct entry to Phase II or Phase III will be suitable and appropriate for therapies that have a human physiological basis or derivation rather than a small molecule, drug or carrier that may be novel and not a known carrier. FDA, Food and Drug Administration; IND, investigational new drug application

In the context of the United States Food and Drug Authority, an intradiscal therapeutic agent will be assessed as a drug product by the Center for Drugs Research and Development (Figure 6). It is expected that a New Drug Application (NDA) has to be lodged, towards which the trial has to be conducted under an IND. This will require a safety combined with dosage study as a Phase II clinical trial followed by an efficacy Phase III trial, where a double blinded randomization (patient and physicians including care team do not know who received the drug till final data analysis) using a placebo arm to compare the experimental therapeutic agent. Since no objective outcome measures are available, the subjectivity of clinical symptoms is high in patients with IVD disease. Therefore, determining the effects of placebo treatment (eg, sham intradiscal injection) is preferable for scientific validity. Placebo treatment creates an ethical dilemma between maximizing the scientific value of the study and minimizing risk to participants. The ethical acceptability of placebo treatment is therefore mainly affected by the associated procedure risks for the relatively healthy patients with IVD disease, which often do not suffer from any comorbidities.^121^ In both Phase II and III trails, data for safety has to be compulsorily obtained and preliminary efficacy can be tested in Phase II. Whether a regulatory agency will accept another expedient clinical trial model like a single arm study without a control will be dependent on the ability of the sponsor to demonstrate compelling socio‐economic reasons or “orphan‐disease” status for their indication. Furthermore, varying dose studies, multiple disc levels treatment or a repeat injection study should best be addressed after market approval for the drug for one level and one dose; as incorporating these questions in a regulatory study will not only make the trial unwieldy, but also add un‐sustainable cost and time. Such further studies can be investigator‐initiated with or without regulatory oversight.

A complete statistical plan and data management plan are essential. The trial has to be listed at clinicaltrials.gov. Data from the IND (in case the therapy is a protein or a drug) has to be submitted for a NDA which requires multiple and stringent regulatory reviews that may include panels consisting of lay persons and experts. Other regulatory regimes have similar or slight variations. In case a scaffold is classified as a device an Investigational Device Exemption (IDE) study submission followed by a Pre Market Approval (PMA) will be needed. The IDE may require a pilot, a pivotal or a comprehensive study based on what is being evaluated and in consultation with the FDA utilizing their pre‐submission process. More importantly, regulatory harmonization between various countries can help to speed up regulatory approvals. Intradiscal therapies that have undergone or are undergoing clinical trials are provided in Table 5. As one can deduce from the table, there are several developed molecular or biological therapies that have failed thus far to show efficacy, while other remain in the race. The challenges and hurdles that still remain may have to do with not only the harsh environment of the degenerative disc. Patient stratification is an important consideration for achieving desirable outcomes. Appropriate trials and identifying which patients will benefit from which treatments is essential to determine the commissioning/approvals of treatment regimes.

**Table 5:** Intradiscal therapies that have undergone or are undergoing clinical trials

Intradiscal therapies under an IND or with a clinical trial number or published			
Active agent	Sponsor name	Clinical trial number/IND/reference	Status/outcome
Allogeneic discogenic cells	DiscGenics Inc.	NCT03347708	FDA allowance of IND to commence clinical study (October 2017). Currently recruiting.
Autologous disc cells	TETEC; NOVOCART	NCT01640457	Phase I/II (*n* = 120); ongoing
Allogeneic juvenile chondrocytes	NuQu, ISTO Technologies Inc.	NCT01771471	Phase II enrolment completed (*n* = 44); final results expected in 2014. Current status: terminated (change in clinical strategy)
Allogeneic mesenchymal precursor cells (MPC‐06‐ID) [STRO enriched]	Mesoblast Ltd.	NCT01290367 NCT02412735 http://mesoblast.com/clinical‐trial‐results/mpc‐06‐id‐phase‐2	Phase II (*n* = 100): significantly greater pain reduction (VAS, ODI, opioid use), but large placebo effect. IVD MRI changes are missing. Phase III (*n* = 360): ongoing. Cells with hyaluronic acid vs placebo
Allogeneic bone marrow‐derived mesenchymal stromal cells	Red de Terapia Celular	NCT01860417	Phase II (*n* = 25): results not published
Allogeneic bone marrow‐derived mesenchymal stromal cells		Noriega et al (2017)^140^	Phase I (*n* = 24) showed safety. 40% responders
Autologous adipose‐derived mesenchymal stromal cells		Kumar et al (2017)^141^	Phase I (*n* = 10) showed safety. VAS and ODI scores significantly improved
Autologous bone marrow concentrate cells		Pettine et al (2015)^142^	Pilot study (*n* = 26): ODI and VAS scores reduced. Eight patients improved by one modified Pfirrmann grade
Placental tissue extract (BioDGenesis)	Semmes‐Murphey Foundation	NCT02379689	Phase I/II (*n* = 30): results unknown
Recombinant human bone morphogenetic protein‐7 (rhBMP‐7)	Stryker; Olympus Biotech	Imai et al (2007)^85^, ^90^	Product available in Australia, Canada, Germany, Italy and Spain for bone formation Development for intradiscal injection did not progress beyond Phase II trials. In line with this, later in vivo experimental work demonstrated the absence of a regenerative effect and possible adverse effects in Beagle dogs
Recombinant human growth and differentiation factor‐5 (rhGDF‐5)	DePuy Synthesis Spine (J&J subsidiary)	NCT01158924 (*n* = 40) NCT00813813 (*n* = 32) NCT01182337 (*n* = 31) NCT01124006 (*n* = 24)	All studies completed Phase II; inconclusive study results
Fibrin	BIOSTAT BIOLOGX	NCT00693784 (pilot study); Yin et al (2014)^143^ NCT01011816 (Phase III)	Pilot study (*n* = 15) showed safety Phase III (*n* = 220) withdrawn because of lack of efficacy Product on market for other indications including surgical hemostasis
Methylene Blue		Peng et al (2010) Levi et al (2014) Kallewaard et al (2016) NTR2547 (NL)^144^, ^145^, ^146^, ^147^	Phase I (*n* = 40): preliminary Peng et al (2010) (*n* = 136): reduction in pain, ODI, improved satisfaction rate Levi et al (2014) (*n* = 16): no clinical success Kallewaard et al (2016) (*n* = 15): 40% positive responders
IL‐6R mAB		Sainoh et al (2017)^148^	Tocilizumab, Actemra, and RoActemra available on market. Efficacy for back pain not known.
Platelet‐rich plasma		NCT02983747 (Phase II) Monfett et al (2016) (literature review) Tuakli‐Wosornu et al (2016) Levi et al (2016) Akeda et al (2017)^149^, ^150^, ^151^, ^152^	Phase II (*n* = 112) completed, results are awaited. Tuakli‐Wosornu et al (2016) (*n* = 47): improvements in FRI, NRS, and patient satisfaction Levi et al (2016) (*n* = 22): after 6 months success rate of 47% (eg, 50% improvement in VAS and 30% decrease in ODI) Akeda et al (2017) (*n* = 14): VAS score improved, MRI T2 values not changed
Glucocorticoid	Hydro‐cortancyl (Predniso‐lone)	NCT00804531 Nguyen et al (2017)^153^	Phase IV (*n* = 137): efficacy not clear
YH14618	Yuhan Corporation	NCT02320019	Peptide derived from biglycan, binds to TGFβ_1_ and downregulates Smad1/5/8 signaling Phase II (*n* = 326) completed, results awaited
AMG0103	AnGes, Inc.	NCT03263611	Nuclear factor‐κB Decoy oligodeoxynucleotide Phase I (*n* = 24): recruiting
SM04690	Samumed LLC	NCT03246399	Small‐molecule inhibitor of Wnt pathway Phase I (*n* = 18): recruiting

Abbreviations: FDA, Food and Drug Administration; FRI, functional rating index; IL‐6 mAB, interleukin‐6 monoclonal antibody; IND, investigational new drug; IVD, intervertebral disc; ODI, Oswestry disability index; OUS, outside of the United States; MRI, magnetic resonance imaging; NRS, numeric rating scale for pain; VAS, visual analog scale.

Funding the intradiscal treatment, will vary in different countries where different medical funding models apply. In some countries where health is mandated both by state and federal/central government, there may be significant variation among states and different payers. Concomitant with the challenges of reimbursement, extensive physician and surgeon education has to be conducted to better understand the indication, technique of delivery, mechanism of action, potential side effects and appropriate follow up of the patients. “More is not always better” as outlined by the well‐known side effects of off‐label use and controversial indication of recombinant Bone Morphogenetic Protein‐2 (BMP‐2) in spinal fusion.^154^ In this respect, cell transplantation and growth factor dosing needs to take into consideration the demanding disc mileau. Once approved, post‐market surveillance and other investigator‐initiated studies will form the basis of systematic reviews and meta‐analysis that will lead to further fine‐tuning of indications and or dose regimes.

## 7 INTELLECTUAL PROPERTY AND COMMERCIAL SUPPORT

Intellectual property (IP) is a term that describes creations of the mind, such as inventions; literary and artistic works; designs; and symbols, names, and images used in commerce. It is divided into industrial property and copyrights. IP protection allows the holder to exclude others from interfering with or using the property right in specified ways. The main forms of IP are patents, copyrights, trademarks, and trade secrets.^155^ To obtain the grant of a patent, one must file a patent application at a patent office. It is important to file an application as soon as possible, because after the filing date, disclosing to the public no longer forms prior art and if a full patent application is granted provides protection for 20 years from the earliest filing date of the application, not from the date that the patent was granted.

The first stage in securing IP is to perform a patent search of databases to find out if a patent has already been filed or granted that is similar to your patent. A great place to start a preliminary patent search is Google patents at https://patents.google.com. Other free patent databases are patent lens https://www.lens.org/lens/bio, DOepatents https://www.osti.gov/doepatents/about.jsp developed by the US Department of Energy (DOE), USPTO (United States Patent and Trademark Office) http://patft.uspto.gov and Espacenet https://worldwide.espacenet.com/advancedSearch?locale=en_EP.

This is important because it is possible to spend considerable funds preparing and filing an application when there is prior art that will prevent a patent, or make the patent very narrow.^156^, ^157^ Those individuals within academic institutions will have a technology transfer office (or equivalent) within their institution who will normally manage this process and provide funding if deemed to have potential. Discussion with the local technology transfer office as soon as inventors feel they have something worth patenting is essential. Most technology transfer offices will have their own experiences of patenting and commercialization who will work with inventors to collect initial patent searches. The knowledge from these searches will help with accentuating both the positive aspects of your invention and the differences that exist over the prior art, leading to a stronger patent application.^157^

The next step is to file for a provisional patent application where the filing date is recorded officially with the assistance of a patent attorney and then within 12 months file a nonprovisional patent application. If inventors fail to submit the full application within 12 months of the provisional patent then this will automatically run out and any protection is lost. While, typically inventors do a search after the filing of the provisional patent application but before the filing of the nonprovisional patent application,^158^ it is advised to complete this prior to filling the provisional. The reason not everyone chooses to do a patent search first is because of the high cost of hiring a patent attorney. Certainly, recording your invention as quickly as possible and getting an early filing date has its advantages. However, the best course to follow, if funds are available, is doing a patent search first before any patent application is filed. By doing a patent search and receiving professional help from a patent attorney you will be able to determine whether it makes sense to move forward and what, if any, rights could be possibly obtained. Furthermore, inventors will search the database for themselves to be informed of the patent landscape so that they can determine whether it even makes sense to start or continue a project in a certain way and whether there may be some available space that they could target.

Do not disclose your inventions until the provisional patent application is on file as any public activity associated with the invention such as telling others at conferences, in abstracts or as a publication negatively affects the patenting.^155^ This creates a conflict with academia in terms of putting students on a patentable project. After all, many academics are going to need assistance from students in order to bring their invention into being. However, students need publications, presentations, abstracts, etc. for their career, which are often delayed by the patenting process.

Some governmental or private funding is only accessible once you have a patent application on file. Typically, the research projects proposed should not only be beneficial to health but also have a strong focus on knowledge translation with clear milestones and decision points. This is also a Catch‐22, that is, easier to get funding with a patent, but need data for the patent, data is usually only possible with funding. Furthermore, once the patent is filed the clock is ticking as the patent is only enforceable for a limited time. This, particularly for clinical therapies, means that protected time can run out before products reach the clinic.

A consideration of where to protect IP is also important, there are many factors to consider before selecting which countries to apply for patent rights.^155^, ^157^ Among the key considerations are the available budget, market opportunities, the time pressure that exist, the location of suppliers and competitors. As a rule of thumb, the most common regions are the United States, Europe, China, Japan, India, Russia, Brazil, South Korea, and Mexico, because they are large economies. Other countries such as Canada, Australia, and South Africa and far eastern economies, such as Thailand and Indonesia, should also be considered. While patent law has been harmonized on an international level, there are still differences between countries. Estimating costs per country per year is challenging. Costs usually have an official, an associate or attorney fee, a translation fee and a maintenance fee in many countries. It is estimated that if one was to obtain a patent in each of the close to 200 countries that exist on the planet the cost would be $2 000 000 for filing, issuance and maintaining the patent for its full term. The best strategy is to first get the US patent on file, then file for a PCT which cost roughly about $5000 and buys you an additional 18 months when you add from the date you file the US patent application before going into other countries or jurisdictions. There are five leading jurisdictions to file patents. The US patent, the European patent office, and the Japan patent office are called the Trilateral and they work closely together. Then there are the Chinese and Korean jurisdictions. Thus 90% of patent applications are filed in the Trilateral, China, and Korea. However, you have to make sure that you have something that is economically worthwhile before going international.

Financing a commercial operation is a difficult task for many academic IP owners, particularly with the added stress of working at a University. In order to encourage commercialization of intellectual property developed by universities or colleges, some types of support and assistance are available. Where technology transfer offices exist, early conversations are essential to gain university backing and support for patent applications and funding. Furthermore, there are often government resources available and nongovernmental organizations that provide support to technology and innovation entrepreneurs. Gaining support for patenting may require development of a business model at many institutions.

Ideas that are promising can start in universities and evolve into highly profitable businesses. The development of a spin‐out from the university is challenging as most academics have no experience in understanding what the customer needs, assessing the demands of the market demand and raising money. Apart from being risky, a spin‐out requires time, effort, and funding. For research to be transformed into a successful business, there are five steps namely, research, proof of research market and technology, market and technology development, product and business development and exit. Funding sources depend on what step and can come from government, holding companies, venture capital, customers, industrial partners, banks, angels and crowd funding to mention a few.

Recently, “incubators” have developed in the United States, Europe, and Asia, where an inventor can have support during product development. These incubators are set‐up and supported by academic institutions, governmental agencies, nonprofit foundations, and even commercial entities with financial support coming from their respective sponsors. Similar to business incubators, these organizations provide not only infrastructure and resources to accelerate business development but to translate the IP towards a commercial product. This is often done in exchange for a share of or exclusive transferrable license rights to the IP. As these incubators or engines can be highly specific to the medical field and hold much specific expertise, they are able to considerably accelerate the formation and maturation of spin‐outs in a successful manner. At the moment it can take millions of dollars and on average 12 years to get from bench to patients depending on the invention.

## 8 DISCUSSION

Over the last two decades, there has been a vast amount of published literature on the preclinical development of novel therapies for IVD degeneration. However, to date we still do not have a therapy which addresses this unmet clinical need. Through scientific research, the IVD field has a greater understanding of the physiological and catabolic environment of the degenerate niche and the biological impact that it has on the cells and extracellular matrix. Research within the community has also uncovered key differences in cell behavior and phenotype with species and culture conditions. It is essential that this scientific knowledge is incorporated into the preclinical design to accelerate and improve translation of findings from preclinical development to clinical studies. It is accepted that the culture conditions used within preliminary proof of concept studies will depend on availability of resources and the research question being investigated. However, at some point during the preclinical phase, novel therapies should be investigated using human cells/tissues within culture conditions that mimic those most closely seen within the degenerate human IVD (Table 1). Utilizing human cells/tissues early within the developmental pipeline will enable aspects such as patient variability to be explored and will require multiple patient samples to be investigated in vitro. These experiments will be the most clinically relevant and thus reduce risk of failure within clinical studies. Standardized approaches to preclinical investigations of novel therapies from 2D culture to organ culture systems, which mimic the in vivo IVD (Figure 1), will aid comparison with competing therapeutics, so that the best candidate therapies go forward to clinical testing and, thus, improve the chances of progression to clinical use. The therapeutics found to be most promising in the preclinical (in vitro*/*ex vivo) phase still need to be tested in animals before they can enter human clinical trials. As no single animal model will exactly match human IVD degeneration in all of its complex aspects, it is important to choose an appropriate animal model, based on the most important research questions and desired outcome parameters. Small animal (eg, rodent) models are very useful for fundamental/safety studies, whereas the more expensive large animal models are more suitable for translational/efficiency studies. In all animal models, however, the outcomes should be determined objectively, for example, by randomization, blinding, and quantitative scoring systems.

Scientists are often not well informed about the regulatory pathway. Hence, they should contact their local regulatory authority as early as possible to gain appropriate advice. To determine the pathway, the therapeutic under investigation should be classified which will define the preclinical work required for regulatory acceptance. As work proceeds, it must be fully documented in accordance with GLP procedures and entered in a treatment strategy file so that they can be submitted and reviewed as part of the regulatory process.

To obtain meaningful results from a clinical trial, regardless of its therapeutic success, applying regulatory knowledge, developing a good design and thorough planning, with sufficient control throughout the trial is essential. Investigator brochures, summarizing principal and pertinent preclinical results, are important to inform the clinical trial team. Clinical trial protocols must define IFU with strict and unambiguous inclusion and exclusion criteria to create a focused and uniform patient population. Outcome measures must be well defined and validated across centers and their collection and management must be controlled.

IP is an almost necessary component for bringing new therapeutic agents to the clinic. Without it, commercialization is nearly impossible. Hence researchers should be mindful of IP in their dissemination activities. IP searches need to be performed early and prior to external discussions. Provisional and patent applications can provide protection, but are not without substantial costs, and a business plan should be developed as part of the IP strategy. For this purpose, discussions with the institutional technology transfer office or patent agents should be done early and are immensely helpful. For IP commercialization, licensing to established companies is one option but SMEs are more attractive in terms of retaining control and involvement. For SMEs, valuable developmental resources include governmental valorization grants/loans and incubators/engines.

In conclusion, for successful translation of regenerative therapies to clinic, it is essential to plan your pathway to clinic from the outset, take advice early on regulatory and commercial aspects and consider the required proof of concept experiments required to satisfy regulatory approval for clinical trials. Do not delay too long, otherwise any protection will run out, which makes commercial interest difficult (and often this is the only way to fund expensive clinical trials).

## References

[1] Bridgelal Ram M , Grocott PR , Weir HC . Issues and challenges of involving users in medical device development. Health Expect. 2008;11:63‐71. 18275403 10.1111/j.1369-7625.2007.00464.x PMC5060429

[2] European Medicine Agency . Good laboratory practice compliance. http://www.ema.europa.eu/ema/index.jsp?curl=pages/regulation/general/general_content_000158.jsp. Accessed April 16, 2018.

[3] Le Maitre CL , Hoyland JA , Freemont AJ . Studies of human intervertebral disc cell function in a constrained in vitro tissue culture system. Spine (Phila Pa 1976). 2004;29:1187‐1195. 15167656 10.1097/00007632-200406010-00006

[4] Paul CP , Zuiderbaan HA , Zandieh Doulabi B , et al. Simulated‐physiological loading conditions preserve biological and mechanical properties of caprine lumbar intervertebral discs in ex vivo culture. PLoS One. 2012;7:e33147. 22427972 10.1371/journal.pone.0033147 PMC3302815

[5] van Dijk B , Potier E , van DIjk M , Langelaan M , Papen‐Botterhuis N , Ito K . Reduced tonicity stimulates an inflammatory response in nucleus pulposus tissue that can be limited by a COX‐2‐specific inhibitor. J Orthop Res. 2015;33:1724‐1731. 25991050 10.1002/jor.22946

[6] Grant M , Epure LM , Salem O , et al. Development of a large animal long‐term intervertebral disc organ culture model that includes the bony vertebrae for ex vivo studies. Tissue Eng Part C Methods. 2016;22:636‐643. 27216856 10.1089/ten.TEC.2016.0049

[7] Rosenzweig DH , Tremblay Gravel J , Bisson D , et al. Comparative analysis in continuous expansion of bovine and human primary nucleus pulposus cells for tissue repair applications. Eur Cell Mater. 2017;33:240‐251. 28345732 10.22203/eCM.v033a18

[8] Bach FC , Laagland LT , Grant MP , et al. Link‐N: the missing link towards intervertebral disc repair is species‐specific. PLoS One. 2017;12:e0187831. 29117254 10.1371/journal.pone.0187831 PMC5679057

[9] Wang F , Gao ZX , Cai F , et al. Formation, function, and exhaustion of notochordal cytoplasmic vacuoles within intervertebral disc: current understanding and speculation. Oncotarget. 2017;8:57800‐57812. 28915712 10.18632/oncotarget.18101 PMC5593684

[10] Bach FC , de Vries SA , Krouwels A , et al. The species‐specific regenerative effects of notochordal cell‐conditioned medium on chondrocyte‐like cells derived from degenerated human intervertebral discs. Eur Cell Mater. 2015;30:132‐147. 26388616 10.22203/ecm.v030a10

[11] Pattappa G , Li Z , Peroglio M , Wismer N , Alini M , Grad S . Diversity of intervertebral disc cells: phenotype and function. J Anat. 2012;221:480‐496. 22686699 10.1111/j.1469-7580.2012.01521.x PMC3512276

[12] Le Maitre CL , Freemont AJ , Hoyland JA . Accelerated cellular senescence in degenerate intervertebral discs: a possible role in the pathogenesis of intervertebral disc degeneration. Arthritis Res Ther. 2007;9:R45. 17498290 10.1186/ar2198 PMC2206356

[13] Vo NV , Hartman RA , Patil PR , et al. Molecular mechanisms of biological aging in intervertebral discs. J Orthop Res. 2016;34:1289‐1306. 26890203 10.1002/jor.23195 PMC4988945

[14] Le Maitre CL , Freemont AJ , Hoyland JA . The role of interleukin‐1 in the pathogenesis of human intervertebral disc degeneration. Arthritis Res Ther. 2005;7:R732‐R745. 15987475 10.1186/ar1732 PMC1175026

[15] Alini M , Eisenstein SM , Ito K , et al. Are animal models useful for studying human disc disorders/degeneration? Eur Spine J. 2008;17:2‐19. 17632738 10.1007/s00586-007-0414-y PMC2365516

[16] Kluba T , Niemeyer T , Gaissmaier C , Grunder T . Human anulus fibrosis and nucleus pulposus cells of the intervertebral disc: effect of degeneration and culture system on cell phenotype. Spine (Phila Pa 1976). 2005;30:2743‐2748. 16371897 10.1097/01.brs.0000192204.89160.6d

[17] Lee JY , Hall R , Pelinkovic D , et al. New use of a three‐dimensional pellet culture system for human intervertebral disc cells: initial characterization and potential use for tissue engineering. Spine (Phila Pa 1976). 2001;26:2316‐2322.11679815

[18] Colombini A , Lopa S , Ceriani C , et al. In vitro characterization and in vivo behavior of human nucleus pulposus and annulus fibrosus cells in clinical‐grade fibrin and collagen‐enriched fibrin gels. Tissue Eng Part A. 2015;21:793‐802. 25236589 10.1089/ten.TEA.2014.0279

[19] Chen JW , Li B , Yang YH , Jiang SD , Jiang LS . Significance of hypoxia in the physiological function of intervertebral disc cells. Crit Rev Eukaryot Gene Expr. 2014;24:193‐204. 25072146 10.1615/critreveukaryotgeneexpr.2014010485

[20] Soukane DM , Shirazi‐Adl A , Urban JP . Analysis of nonlinear coupled diffusion of oxygen and lactic acid in intervertebral discs. J Biomech Eng. 2005;127:1121‐1126. 16502654 10.1115/1.2073674

[21] Mokhbi Soukane D , Shirazi‐Adl A , Urban JP . Investigation of solute concentrations in a 3D model of intervertebral disc. Eur Spine J. 2009;18:254‐262. 10.1007/s00586-008-0822-7 PMC2899342 19015897

[22] Kraemer J , Kolditz D , Gowin R . Water and electrolyte content of human intervertebral discs under variable load. Spine (Phila Pa 1976). 1985;10:69‐71. 3983704 10.1097/00007632-198501000-00011

[23] Li H , Liang C , Tao Y , et al. Acidic pH conditions mimicking degenerative intervertebral discs impair the survival and biological behavior of human adipose‐derived mesenchymal stem cells. Exp Biol Med (Maywood). 2012;237:845‐852. 22829705 10.1258/ebm.2012.012009

[24] Kitano T , Zerwekh JE , Usui Y , Edwards ML , Flicker PL , Mooney V . Biochemical changes associated with the symptomatic human intervertebral disk. Clin Orthop Relat Res. 1993;293:372‐377. 8339506

[25] Grant MP , Epure LM , Bokhari R , et al. Human cartilaginous endplate degeneration is induced by calcium and the extracellular calcium‐sensing receptor in the intervertebral disc. Eur Cell Mater. 2016;32:137‐151. 27452962 10.22203/ecm.v032a09

[26] Phillips KL , Cullen K , Chiverton N , et al. Potential roles of cytokines and chemokines in human intervertebral disc degeneration: Interleukin‐1 is a master regulator of catabolic processes. Osteoarthritis Cartilage. 2015;23:1165‐1177. 25748081 10.1016/j.joca.2015.02.017

[27] Arkesteijn IT , Mouser VH , Mwale F , van Dijk BG , Ito K . A well‐controlled nucleus pulposus tissue culture system with injection port for evaluating regenerative therapies. Ann Biomed Eng. 2016;44:1798‐1807. 26294008 10.1007/s10439-015-1428-y PMC4837215

[28] van Dijk BG , Potier E , Ito K . Long‐term culture of bovine nucleus pulposus explants in a native environment. Spine J. 2013;13:454‐463. 23340344 10.1016/j.spinee.2012.12.006

[29] van Dijk B , Potier E , Ito K . Culturing bovine nucleus pulposus explants by balancing medium osmolarity. Tissue Eng Part C Methods. 2011;17:1089‐1096. 21718168 10.1089/ten.TEC.2011.0215

[30] Gantenbein B , Grunhagen T , Lee CR , et al. An in vitro organ culturing system for intervertebral disc explants with vertebral endplates: a feasibility study with ovine caudal discs. Spine (Phila Pa 1976). 2006;31:2665‐2673. 17077734 10.1097/01.brs.0000244620.15386.df

[31] van Dijk B , Potier E , Licht R , Creemers L , Ito K . The effect of a cyclooxygenase 2 inhibitor on early degenerated human nucleus pulposus explants. Global Spine J. 2014;4:33‐40. 24494179 10.1055/s-0033-1359724 PMC3908972

[32] van Dijk BGM , Potier E , van Dijk M , Creemers LB , Ito K . Osteogenic protein 1 does not stimulate a regenerative effect in cultured human degenerated nucleus pulposus tissue. J Tissue Eng Regen Med. 2017;11:2127‐2135. 26612824 10.1002/term.2111

[33] Zhang Y , Phillips FM , Thonar EJ , et al. Cell therapy using articular chondrocytes overexpressing BMP‐7 or BMP‐10 in a rabbit disc organ culture model. Spine (Phila Pa 1976). 2008;33:831‐838. 18404100 10.1097/BRS.0b013e31816b1f38

[34] Le Maitre CL , Baird P , Freemont AJ , Hoyland JA . An in vitro study investigating the survival and phenotype of mesenchymal stem cells following injection into nucleus pulposus tissue. Arthritis Res Ther. 2009;11:R20. 19210770 10.1186/ar2611 PMC2688252

[35] Arkesteijn ITM , Potier E , Ito K . The regenerative potential of notochordal cells in a nucleus pulposus explant. Global Spine J. 2017;7:14‐20. 28451504 10.1055/s-0036-1583174 PMC5400162

[36] Thorpe AA , Dougill G , Vickers L , et al. Thermally triggered hydrogel injection into bovine intervertebral disc tissue explants induces differentiation of mesenchymal stem cells and restores mechanical function. Acta Biomater. 2017;54:212‐226. 28285075 10.1016/j.actbio.2017.03.010

[37] AlGarni N , Grant MP , Epure LM , et al. Short link N stimulates intervertebral disc repair in a novel long‐term organ culture model that includes the bony vertebrae. Tissue Eng Part A. 2016;22:1252‐1257. 27673512 10.1089/ten.TEA.2016.0115

[38] Walter BA , Illien‐Junger S , Nasser PR , Hecht AC , Iatridis JC . Development and validation of a bioreactor system for dynamic loading and mechanical characterization of whole human intervertebral discs in organ culture. J Biomech. 2014;47:2095‐2101. 24725441 10.1016/j.jbiomech.2014.03.015 PMC4047158

[39] Gawri R , Mwale F , Ouellet J , et al. Development of an organ culture system for long‐term survival of the intact human intervertebral disc. Spine (Phila Pa 1976). 2011;36:1835‐1842. 21270705 10.1097/BRS.0b013e3181f81314

[40] Parolin M , Gawri R , Mwale F , et al. Development of a whole disc organ culture system to study human intervertebral disc. Evid Based Spine Care J. 2010;1:67‐68. 10.1055/s-0028-1100919 PMC3623092 23637672

[41] Gantenbein B , Illien‐Junger S , Chan SC , et al. Organ culture bioreactors‐platforms to study human intervertebral disc degeneration and regenerative therapy. Curr Stem Cell Res Ther. 2015;10:339‐352. 25764196 10.2174/1574888x10666150312102948 PMC4437861

[42] Peroglio M , Gaspar D , Zeugolis DI , Alini M . Relevance of bioreactors and whole tissue cultures for the translation of new therapies to humans. J Orthop Res. 2018;36(1):10‐21. 28718947 10.1002/jor.23655

[43] Sakai D , Andersson GB . Stem cell therapy for intervertebral disc regeneration: obstacles and solutions. Nat Rev Rheumatol. 2015;11:243‐256. 25708497 10.1038/nrrheum.2015.13

[44] Selard E , Shirazi‐Adl A , Urban JP . Finite element study of nutrient diffusion in the human intervertebral disc. Spine (Phila Pa 1976). 2003;28:1945‐1953. 12973139 10.1097/01.BRS.0000087210.93541.23

[45] Pei M . Environmental preconditioning rejuvenates adult stem cells' proliferation and chondrogenic potential. Biomaterials. 2017;117:10‐23. 27923196 10.1016/j.biomaterials.2016.11.049 PMC5177456

[46] Krouwels A , Melchels FPW , van Rijen MHP , et al. Comparing hydrogels for human nucleus pulposus cell regeneration: role of osmolarity during expansion. Tissue Eng Part C Methods. 2018;24(4):222‐232. 29457534 10.1089/ten.TEC.2017.0226

[47] Bibby SR , Jones DA , Ripley RM , Urban JP . Metabolism of the intervertebral disc: effects of low levels of oxygen, glucose, and pH on rates of energy metabolism of bovine nucleus pulposus cells. Spine (Phila Pa 1976). 2005;30:487‐496. 15738779 10.1097/01.brs.0000154619.38122.47

[48] Le Maitre CL , Binch AL , Thorpe AA , Hughes SP . Degeneration of the intervertebral disc with new approaches for treating low back pain. J Neurosurg Sci. 2015;59:47‐61. 25423135

[49] Sakai D , Schol J . Cell therapy for intervertebral disc repair: clinical perspective. J Orthop Translat. 2017;9:8‐18. 29662795 10.1016/j.jot.2017.02.002 PMC5822958

[50] Roberts S , Menage J , Sivan S , Urban JP . Bovine explant model of degeneration of the intervertebral disc. BMC Musculoskelet Disord. 2008;9:24. 18298830 10.1186/1471-2474-9-24 PMC2266744

[51] Korecki CL , Costi JJ , Iatridis JC . Needle puncture injury affects intervertebral disc mechanics and biology in an organ culture model. Spine (Phila Pa 1976). 2008;33:235‐241. 18303454 10.1097/BRS.0b013e3181624504 PMC2587060

[52] Li Z , Lezuo P , Pattappa G , et al. Development of an ex vivo cavity model to study repair strategies in loaded intervertebral discs. Eur Spine J. 2016;25:2898‐2908. 27037921 10.1007/s00586-016-4542-0

[53] Gu W , Zhu Q , Gao X , Brown MD . Simulation of the progression of intervertebral disc degeneration due to decreased nutritional supply. Spine (Phila Pa 1976). 2014;39:E1411‐E1417. 25188596 10.1097/BRS.0000000000000560 PMC4229452

[54] Zhu Q , Gao X , Temple HT , Brown MD , Gu W . Simulation of biological therapies for degenerated intervertebral discs. J Orthop Res. 2016;34:699‐708. 26425965 10.1002/jor.23061 PMC4833445

[55] Schmidt H , Galbusera F , Rohlmann A , Shirazi‐Adl A . What have we learned from finite element model studies of lumbar intervertebral discs in the past four decades? J Biomech. 2013;46:2342‐2355. 23962527 10.1016/j.jbiomech.2013.07.014

[56] Viceconti M , Henney A , Morley‐Fletcher E . In silico clinical trials: how computer simulation will transform the biomedical industry. Int J Clin Trials. 2016;3(2):37‐46.

[57] Le Maitre CL , Pockert A , Buttle DJ , Freemont AJ , Hoyland JA . Matrix synthesis and degradation in human intervertebral disc degeneration. Biochem Soc Trans. 2007;35:652‐655. 17635113 10.1042/BST0350652

[58] Roughley PJ . Biology of intervertebral disc aging and degeneration: involvement of the extracellular matrix. Spine (Phila Pa 1976). 2004;29:2691‐2699. 15564918 10.1097/01.brs.0000146101.53784.b1

[59] Brinjikji W , Diehn FE , Jarvik JG , et al. MRI findings of disc degeneration are more prevalent in adults with low back pain than in asymptomatic controls: a systematic review and meta‐analysis. AJNR Am J Neuroradiol. 2015;36:2394‐2399. 26359154 10.3174/ajnr.A4498 PMC7964277

[60] Brinjikji W , Luetmer PH , Comstock B , et al. Systematic literature review of imaging features of spinal degeneration in asymptomatic populations. AJNR Am J Neuroradiol. 2015;36:811‐816. 25430861 10.3174/ajnr.A4173 PMC4464797

[61] Pye SR , Reid DM , Smith R , et al. Radiographic features of lumbar disc degeneration and self‐reported back pain. J Rheumatol. 2004;31:753‐758. 15088303

[62] Adams MA . Biomechanics of back pain. Acupunct Med. 2004;22:178‐188. 15628775 10.1136/aim.22.4.178

[63] Peng B , Hao J , Hou S , et al. Possible pathogenesis of painful intervertebral disc degeneration. Spine (Phila Pa 1976). 2006;31:560‐566. 16508552 10.1097/01.brs.0000201324.45537.46

[64] Shi C , Qiu S , Riester SM , et al. Animal models for studying the etiology and treatment of low back pain. J Orthop Res. 2018;36(5):1305‐1312. 28921656 10.1002/jor.23741 PMC6287742

[65] Meij BP , Bergknut N . Degenerative lumbosacral stenosis in dogs. Vet Clin North Am Small Anim Pract. 2010;40:983‐1009. 20732601 10.1016/j.cvsm.2010.05.006

[66] Tellegen AR , Willems N , Tryfonidou MA , Meij BP . Pedicle screw‐rod fixation: a feasible treatment for dogs with severe degenerative lumbosacral stenosis. BMC Vet Res. 2015;11:299. 26642756 10.1186/s12917-015-0614-3 PMC4672470

[67] Tellegen AR , Willems N , Beukers M , et al. Intradiscal application of a PCLA‐PEG‐PCLA hydrogel loaded with celecoxib for the treatment of back pain in canines: What's in it for humans? J Tissue Eng Regen Med. 2017;12(3):642‐652. 28544701 10.1002/term.2483

[68] Stolworthy DK , Fullwood RA , Merrell TM , Bridgewater LC , Bowden AE . Biomechanical analysis of the camelid cervical intervertebral disc. J Orthopaedic Translation. 2015;3(1):34‐43. 10.1016/j.jot.2014.12.001 PMC5982393 30035038

[69] Bowden AE , Stolworthy DK , Fullwood RA , Bridgewater LC . Mechanical parallels for a camelid cervical spine model of lumbar disc degeneration. Conference Paper presented at: 2nd International Spine Research Symposium, 2013; Philadelphia, PA.

[70] Bergknut N , Smolders LA , Grinwis GC , et al. Intervertebral disc degeneration in the dog. Part 1: anatomy and physiology of the intervertebral disc and characteristics of intervertebral disc degeneration. Vet J. 2013;195:282‐291. 23177522 10.1016/j.tvjl.2012.10.024

[71] Chan D , Song Y , Sham P , Cheung KM . Genetics of disc degeneration. Eur Spine J. 2006;15(suppl 3):S317‐S325. 16819621 10.1007/s00586-006-0171-3 PMC2335375

[72] Feng Y , Egan B , Wang J . Genetic factors in intervertebral disc degeneration. Genes Dis. 2016;3:178‐185. 27617275 10.1016/j.gendis.2016.04.005 PMC5016799

[73] Kamper M , Hamann N , Prein C , et al. Early changes in morphology, bone mineral density and matrix composition of vertebrae lead to disc degeneration in aged collagen IX −/− mice. Matrix Biol. 2016;49:132‐143. 26429145 10.1016/j.matbio.2015.09.005

[74] Zhang Y , Xiong C , Kudelko M , et al. Early onset of disc degeneration in SM/J mice is associated with changes in ion transport systems and fibrotic events. Matrix Biol. 2018 10.1016/j.matbio.2018.03.024 29649547

[75] Choi H , Tessier S , Silagi ES , et al. A novel mouse model of intervertebral disc degeneration shows altered cell fate and matrix homeostasis. Matrix Biol. 2018 10.1016/j.matbio.2018.03.019 PMC6081256 29605718

[76] Bach FC , Zhang Y , Miranda‐Bedate A , et al. Increased caveolin‐1 in intervertebral disc degeneration facilitates repair. Arthritis Res Ther. 2016;18:59. 26939667 10.1186/s13075-016-0960-y PMC4778307

[77] Brown EA , Dickinson PJ , Mansour T , et al. FGF4 retrogene on CFA12 is responsible for chondrodystrophy and intervertebral disc disease in dogs. Proc Natl Acad Sci U S A. 2017;114:11476‐11481. 29073074 10.1073/pnas.1709082114 PMC5664524

[78] Mao HJ , Chen QX , Han B , et al. The effect of injection volume on disc degeneration in a rat tail model. Spine (Phila Pa 1976). 2011;36:E1062‐E1069. 21358491 10.1097/BRS.0b013e3182027d42

[79] Carragee EJ , Don AS , Hurwitz EL , Cuellar JM , Carrino J , Herzog R . 2009 ISSLS prize winner: does discography cause accelerated progression of degeneration changes in the lumbar disc: a ten‐year matched cohort study. Spine (Phila Pa 1976). 2009;34:2338‐2345. 19755936 10.1097/BRS.0b013e3181ab5432

[80] Matta A , Karim MZ , Isenman DE , Erwin WM . Molecular therapy for degenerative disc disease: clues from secretome analysis of the notochordal cell‐rich nucleus pulposus. Sci Rep. 2017;7:45623. 28358123 10.1038/srep45623 PMC5372366

[81] Kawakami M , Matsumoto T , Hashizume H , Kuribayashi K , Chubinskaya S , Yoshida M . Osteogenic protein‐1 (osteogenic protein‐1/bone morphogenetic protein‐7) inhibits degeneration and pain‐related behavior induced by chronically compressed nucleus pulposus in the rat. Spine (Phila Pa 1976). 2005;30:1933‐1939. 16135982 10.1097/01.brs.0000176319.78887.64

[82] Zhang H , Wang L , Park JB , et al. Intradiscal injection of simvastatin retards progression of intervertebral disc degeneration induced by stab injury. Arthritis Res Ther. 2009;11:R172. 19912653 10.1186/ar2861 PMC3003500

[83] Masuda K , Imai Y , Okuma M , et al. Osteogenic protein‐1 injection into a degenerated disc induces the restoration of disc height and structural changes in the rabbit anular puncture model. Spine (Phila Pa 1976). 2006;31:742‐754. 16582847 10.1097/01.brs.0000206358.66412.7b

[84] Miyamoto K , Masuda K , Kim JG , et al. Intradiscal injections of osteogenic protein‐1 restore the viscoelastic properties of degenerated intervertebral discs. Spine J. 2006;6:692‐703. 17088200 10.1016/j.spinee.2006.04.014

[85] Imai Y , Okuma M , An HS , et al. Restoration of disc height loss by recombinant human osteogenic protein‐1 injection into intervertebral discs undergoing degeneration induced by an intradiscal injection of chondroitinase ABC. Spine (Phila Pa 1976). 2007;32:1197‐1205. 17495776 10.1097/BRS.0b013e3180574d26

[86] An HS , Takegami K , Kamada H , et al. Intradiscal administration of osteogenic protein‐1 increases intervertebral disc height and proteoglycan content in the nucleus pulposus in normal adolescent rabbits. Spine (Phila Pa 1976). 2005;30:25‐32. 15626976 10.1097/01.brs.0000148002.68656.4d

[87] Chujo T , An HS , Akeda K , et al. Effects of growth differentiation factor‐5 on the intervertebral disc—in vitro bovine study and in vivo rabbit disc degeneration model study. Spine (Phila Pa 1976). 2006;31:2909‐2917. 17139221 10.1097/01.brs.0000248428.22823.86

[88] Mwale F , Masuda K , Pichika R , et al. The efficacy of link N as a mediator of repair in a rabbit model of intervertebral disc degeneration. Arthritis Res Ther. 2011;13:R120. 21787415 10.1186/ar3423 PMC3239358

[89] Willems N , Mihov G , Grinwis GC , et al. Safety of intradiscal injection and biocompatibility of polyester amide microspheres in a canine model predisposed to intervertebral disc degeneration. J Biomed Mater Res B Appl Biomater. 2017;105:707‐714. 26687460 10.1002/jbm.b.33579 PMC6690078

[90] Willems N , Bach FC , Plomp SG , et al. Intradiscal application of rhBMP‐7 does not induce regeneration in a canine model of spontaneous intervertebral disc degeneration. Arthritis Res Ther. 2015;17:137. 26013758 10.1186/s13075-015-0625-2 PMC4443547

[91] Benz K , Stippich C , Fischer L , et al. Intervertebral disc cell‐ and hydrogel‐supported and spontaneous intervertebral disc repair in nucleotomized sheep. Eur Spine J. 2012;21:1758‐1768. 22842955 10.1007/s00586-012-2443-4 PMC3459128

[92] Freeman BJ , Kuliwaba JS , Jones CF , et al. Allogeneic mesenchymal precursor cells promote healing in postero‐lateral annular lesions and improve indices of lumbar intervertebral disc degeneration in an ovine model. Spine (Phila Pa 1976). 2016;41:1331‐1339. 26913464 10.1097/BRS.0000000000001528

[93] Vadala G , Russo F , Pattappa G , et al. The transpedicular approach as an alternative route for intervertebral disc regeneration. Spine (Phila Pa 1976). 2013;38:E319‐E324. 23324932 10.1097/BRS.0b013e318285bc4a

[94] Le Fournier L , Fusellier M , Halgand B , et al. The transpedicular surgical approach for the development of intervertebral disc targeting regenerative strategies in an ovine model. Eur Spine J. 2017;26:2072‐2083. 28674787 10.1007/s00586-017-5199-z

[95] Gruber HE , Phillips R , Ingram JA , Norton HJ , Hanley EN Jr . Spontaneous age‐related cervical disc degeneration in the sand rat. Clin Orthop Relat Res. 2014;472:1936‐1942. 24515407 10.1007/s11999-014-3497-x PMC4016433

[96] Moskowitz RW , Ziv I , Denko CW , Boja B , Jones PK , Adler JH . Spondylosis in sand rats: a model of intervertebral disc degeneration and hyperostosis. J Orthop Res. 1990;8:401‐411. 2182801 10.1002/jor.1100080312

[97] Smolders LA , Bergknut N , Grinwis GC , et al. Intervertebral disc degeneration in the dog. Part 2: Chondrodystrophic and non‐chondrodystrophic breeds. Vet J. 2013;195:292‐299. 23154070 10.1016/j.tvjl.2012.10.011

[98] Beierfuss A , Dietrich H , Kremser C , et al. Knockout of apolipoprotein E in rabbit promotes premature intervertebral disc degeneration: a new in vivo model for therapeutic approaches of spinal disc disorders. PLoS One. 2017;12:e0187564. 29099857 10.1371/journal.pone.0187564 PMC5669473

[99] Sakai D , Mochida J , Iwashina T , et al. Regenerative effects of transplanting mesenchymal stem cells embedded in atelocollagen to the degenerated intervertebral disc. Biomaterials. 2006;27:335‐345. 16112726 10.1016/j.biomaterials.2005.06.038

[100] Chen CH , Chiang CJ , Wu LC , et al. Time course investigation of intervertebral disc degeneration in a rat‐tail puncture model. Life Sci. 2016;156:15‐20. 27197027 10.1016/j.lfs.2016.05.020

[101] Tian Z , Ma X , Yasen M , et al. Intervertebral disc degeneration in a percutaneous mouse tail injury model. Am J Phys Med Rehabil. 2018;97:170‐177. 28863006 10.1097/PHM.0000000000000818 PMC5823709

[102] Zhang H , La Marca F , Hollister SJ , Goldstein SA , Lin CY . Developing consistently reproducible intervertebral disc degeneration at rat caudal spine by using needle puncture. J Neurosurg Spine. 2009;10:522‐530. 19558284 10.3171/2009.2.SPINE08925

[103] Yan Z , Pan Y , Wang S , et al. Static compression induces ECM remodeling and integrin alpha2beta1 expression and signaling in a rat tail caudal intervertebral disc degeneration model. Spine (Phila Pa 1976). 2017;42:E448‐E458. 27548579 10.1097/BRS.0000000000001856

[104] Miyagi M , Ishikawa T , Kamoda H , et al. ISSLS prize winner: disc dynamic compression in rats produces long‐lasting increases in inflammatory mediators in discs and induces long‐lasting nerve injury and regeneration of the afferent fibers innervating discs: a pathomechanism for chronic discogenic low back pain. Spine (Phila Pa 1976). 2012;37:1810‐1818. 22366969 10.1097/BRS.0b013e31824ffac6

[105] Sakai D , Nishimura K , Tanaka M , et al. Migration of bone marrow‐derived cells for endogenous repair in a new tail‐looping disc degeneration model in the mouse: a pilot study. Spine J. 2015;15:1356‐1365. 25459743 10.1016/j.spinee.2013.07.491

[106] McCann MR , Veras MA , Yeung C , et al. Whole‐body vibration of mice induces progressive degeneration of intervertebral discs associated with increased expression of il‐1beta and multiple matrix degrading enzymes. Osteoarthritis Cartilage. 2017;25:779‐789. 28104539 10.1016/j.joca.2017.01.004

[107] Obata S , Akeda K , Imanishi T , et al. Effect of autologous platelet‐rich plasma‐releasate on intervertebral disc degeneration in the rabbit anular puncture model: a preclinical study. Arthritis Res Ther. 2012;14:R241. 23127251 10.1186/ar4084 PMC3674597

[108] Xin L , Xu W , Yu L , et al. Effects of annulus defects and implantation of poly(lactic‐co‐glycolic acid) (PLGA)/fibrin gel scaffolds on nerves ingrowth in a rabbit model of annular injury disc degeneration. J Orthop Surg Res. 2017;12:73. 28499451 10.1186/s13018-017-0572-5 PMC5429511

[109] Hiyama A , Mochida J , Iwashina T , et al. Transplantation of mesenchymal stem cells in a canine disc degeneration model. J Orthop Res. 2008;26:589‐600. 18203202 10.1002/jor.20584

[110] Bach FC , Tellegen AR , Beukers M , et al. Biologic canine and human intervertebral disc repair by notochordal cell‐derived matrix: from bench towards bedside. Oncotarget. 2018;9:26507‐26526. 29899873 10.18632/oncotarget.25476 PMC5995168

[111] Fusellier M , Colombier P , Lesoeur J , et al. Longitudinal comparison of enzyme‐ and laser‐treated intervertebral disc by MRI, X‐ray, and histological analyses reveals discrepancies in the progression of disc degeneration: a rabbit study. Biomed Res Int. 2016;2016:1‐12. 10.1155/2016/5498271 PMC4877459 27247937

[112] Hunter CJ , Matyas JR , Duncan NA . The notochordal cell in the nucleus pulposus: a review in the context of tissue engineering. Tissue Eng. 2003;9:667‐677. 13678445 10.1089/107632703768247368

[113] Lotz JC , Colliou OK , Chin JR , Duncan NA , Liebenberg E . Compression‐induced degeneration of the intervertebral disc: an in vivo mouse model and finite‐element study. Spine (Phila Pa 1976). 1998;23:2493‐2506. 9854748 10.1097/00007632-199812010-00004

[114] Iatridis JC , Mente PL , Stokes IA , Aronsson DD , Alini M . Compression‐induced changes in intervertebral disc properties in a rat tail model. Spine (Phila Pa 1976). 1999;24:996‐1002. 10332792 10.1097/00007632-199905150-00013

[115] Anderson DG , Izzo MW , Hall DJ , et al. Comparative gene expression profiling of normal and degenerative discs: analysis of a rabbit annular laceration model. Spine (Phila Pa 1976). 2002;27:1291‐1296. 12065976 10.1097/00007632-200206150-00009

[116] Kadoya K , Kotani Y , Abumi K , et al. Biomechanical and morphologic evaluation of a three‐dimensional fabric sheep artificial intervertebral disc: in vitro and in vivo analysis. Spine (Phila Pa 1976). 2001;26:1562‐1569. 11462087 10.1097/00007632-200107150-00012

[117] Hoogendoorn R , Doulabi BZ , Huang CL , Wuisman PI , Bank RA , Helder MN . Molecular changes in the degenerated goat intervertebral disc. Spine (Phila Pa 1976). 2008;33:1714‐1721. 18628703 10.1097/BRS.0b013e31817d2468

[118] Smit TH . The use of a quadruped as an in vivo model for the study of the spine—biomechanical considerations. Eur Spine J. 2002;11:137‐144. 11956920 10.1007/s005860100346 PMC3610505

[119] Wilke HJ , Kettler A , Claes LE . Are sheep spines a valid biomechanical model for human spines? Spine (Phila Pa 1976). 1997;22:2365‐2374. 9355217 10.1097/00007632-199710150-00009

[120] Bergknut N , Rutges JP , Kranenburg HJ , et al. The dog as an animal model for intervertebral disc degeneration? Spine (Phila Pa 1976). 2012;37:351‐358. 21544011 10.1097/BRS.0b013e31821e5665

[121] Niemansburg SL , van Delden JJ , Dhert WJ , Bredenoord AL . Regenerative medicine interventions for orthopedic disorders: ethical issues in the translation into patients. Regen Med. 2013;8:65‐73. 23259806 10.2217/rme.12.112

[122] Tam V , Chan WCW , Leung VYL , et al. Histological and reference system for the analysis of mouse intervertebral disc. J Orthop Res. 2018;6(1):233‐224. 10.1002/jor.23637 28636254

[123] Bergknut N , Meij BP , Hagman R , et al. Intervertebral disc disease in dogs. Part 1: a new histological grading scheme for classification of intervertebral disc degeneration in dogs. Vet J. 2013;195:156‐163. 22789628 10.1016/j.tvjl.2012.05.027

[124] Nisolle JF , Bihin B , Kirschvink N , et al. Prevalence of age‐related changes in ovine lumbar intervertebral discs during computed tomography and magnetic resonance imaging. Comp Med. 2016;66:300‐307. 27538861 PMC4983172

[125] Hebelka H , Nilsson A , Ekstrom L , Hansson T . In vivo discography in degenerate porcine spines revealed pressure transfer to adjacent discs. Spine (Phila Pa 1976). 2013;38:E1575‐E1582. 24296483 10.1097/01.brs.0000435141.61593.05

[126] Johannessen W , Auerbach JD , Wheaton AJ , et al. Assessment of human disc degeneration and proteoglycan content using T1rho‐weighted magnetic resonance imaging. Spine (Phila Pa 1976). 2006;31:1253‐1257. 16688040 10.1097/01.brs.0000217708.54880.51 PMC2855820

[127] Ogon I , Takebayashi T , Takashima H , et al. Analysis of chronic low back pain with magnetic resonance imaging T2 mapping of lumbar intervertebral disc. J Orthop Sci. 2015;20:295‐301. 25649736 10.1007/s00776-014-0686-0

[128] Dudli S , Fields AJ , Samartzis D , Karppinen J , Lotz JC . Pathobiology of modic changes. Eur Spine J. 2016;25:3723‐3734. 26914098 10.1007/s00586-016-4459-7 PMC5477843

[129] Luoma K , Vehmas T , Kerttula L , Gronblad M , Rinne E . Chronic low back pain in relation to modic changes, bony endplate lesions, and disc degeneration in a prospective MRI study. Eur Spine J. 2016;25:2873‐2881. 27480265 10.1007/s00586-016-4715-x

[130] Institute of Medicine . Medical Devices and the public's health—the FDA 510(k) clearance process at 35 years, 2011 http://www.nationalacademies.org/hmd/Reports/2011/Medical‐Devices‐and‐the‐Publics‐Health‐The‐FDA‐510k‐Clearance‐Process‐at‐35‐Years.aspx. Accessed XX X, XX.

[131] US Food & Drug Administration FDA 101: regulating biological products. https://www.fda.gov/ForConsumers/ConsumerUpdates/ucm048341.htm. Accessed XX X, XX.

[132] European Medicines Agency . ICH topic Q 5 E comparability of biotechnological/biological products/Note for guidance on biotechnological/biological products subject to changes in their manufacturing process (CPMP/ICH/5721/03), 2005 http://www.ema.europa.eu/docs/en_GB/document_library/Scientific_guideline/2009/09/WC500002805.pdf. Accessed XX X, XX.

[133] Thorpe AA , Sammon C , Le Maitre CL . ‘Cell or not to cell’ that is the question: for intervertebral disc regeneration? J Stem Cell Res Dev. 2015;2:1.

[134] Bowles RD , Setton LA . Biomaterials for intervertebral disc regeneration and repair. Biomaterials. 2017;129:54‐67. 28324865 10.1016/j.biomaterials.2017.03.013 PMC5627607

[135] European Commission . Medical devices: guidance document—borderline products, drug‐delivery products and medical devices incorporating, as an integral part, an ancillary medicinal substance or an ancillary human blood derivative. http://www.gpc.center/download/807. Accessed XX X, XX.

[136] ISO 10993‐1:2009. Biological evaluation of medical devices. Part 1: evaluation and testing within a risk manage process. https://www.iso.org/standard/44908.html. Accessed XX X, XX.

[137] European Parlement . Regulation (EC) no. 1394/2007 of the European parliament and of the council on advanced therapy medicinal products and amending directive 2001/83/EC and regulation (EC) no 726/2004, 2007 Official Journal of the European Union. https://ec.europa.eu/health/sites/health/files/files/eudralex/vol-1/reg_2007_1394/reg_2007_1394_en.pdf. Accessed XX X, XX.

[138] LiArno S , Khayatzadeh S , Hamilton D , Bini S , Chahine N . Outcome measures in orthopaedic research of the joints and spine. Scientific Workshop at the Proceedings of the Annual Scientific Meeting of the Orthopaedic Research Society, New Orleans, USA, 2018.

[139] Samartzis D , Borthakur A , Belfer I , et al. Novel diagnostic and prognostic methods for disc degeneration and low back pain. Spine J. 2015;15:1919‐1932. 26303178 10.1016/j.spinee.2014.09.010 PMC5473425

[140] Noriega DC , Ardura F , Hernandez‐Ramajo R , et al. Intervertebral disc repair by allogeneic mesenchymal bone marrow cells: a randomized controlled trial. Transplantation. 2017;101:1945‐1951. 27661661 10.1097/TP.0000000000001484

[141] Kumar H , Ha DH , Lee EJ , et al. Safety and tolerability of intradiscal implantation of combined autologous adipose‐derived mesenchymal stem cells and hyaluronic acid in patients with chronic discogenic low back pain: 1‐year follow‐up of a phase I study. Stem Cell Res Ther. 2017;8:262. 29141662 10.1186/s13287-017-0710-3 PMC5688755

[142] Pettine KA , Murphy MB , Suzuki RK , Sand TT . Percutaneous injection of autologous bone marrow concentrate cells significantly reduces lumbar discogenic pain through 12 months. Stem Cells. 2015;33:146‐156. 25187512 10.1002/stem.1845

[143] Yin W , Pauza K , Olan WJ , Doerzbacher JF , Thorne KJ . Intradiscal injection of fibrin sealant for the treatment of symptomatic lumbar internal disc disruption: results of a prospective multicenter pilot study with 24‐month follow‐up. Pain Med. 2014;15:16‐31. 24152079 10.1111/pme.12249

[144] Kallewaard JW , Geurts JW , Kessels A , Willems P , van Santbrink H , van Kleef M . Efficacy, safety, and predictors of intradiscal methylene blue injection for discogenic low back pain: results of a multicenter prospective clinical series. Pain Pract. 2016;16:405‐412. 25753429 10.1111/papr.12283

[145] Geurts JW , Kallewaard JW , Kessels A , et al. Efficacy and cost‐effectiveness of intradiscal methylene blue injection for chronic discogenic low back pain: study protocol for a randomized controlled trial. Trials. 2015;16:532. 26590962 10.1186/s13063-015-1058-6 PMC4654797

[146] Peng B , Pang X , Wu Y , Zhao C , Song X . A randomized placebo‐controlled trial of intradiscal methylene blue injection for the treatment of chronic discogenic low back pain. Pain. 2010;149:124‐129. 20167430 10.1016/j.pain.2010.01.021

[147] Levi DS , Horn S , Walko E . Intradiskal methylene blue treatment for diskogenic low back pain. PM R. 2014;6:1030‐1037. 24780850 10.1016/j.pmrj.2014.04.008

[148] Sainoh T , Inage K , Orita S , et al. Correlation among inflammatory cytokine expression levels, degree of disk degeneration, and predominant clinical symptoms in patients with degenerated intervertebral discs. Asian Spine J. 2017;11:472‐477. 28670416 10.4184/asj.2017.11.3.472 PMC5481603

[149] Monfett M , Harrison J , Boachie‐Adjei K , Lutz G . Intradiscal platelet‐rich plasma (PRP) injections for discogenic low back pain: an update. Int Orthop. 2016;40:1321‐1328. 27073034 10.1007/s00264-016-3178-3

[150] Tuakli‐Wosornu YA , Terry A , Boachie‐Adjei K , et al. Lumbar intradiskal platelet‐rich plasma (PRP) injections: a prospective, double‐blind, randomized controlled study. PM R. 2016;8:1‐10. 26314234 10.1016/j.pmrj.2015.08.010

[151] Levi D , Horn S , Tyszko S , Levin J , Hecht‐Leavitt C , Walko E . Intradiscal platelet‐rich plasma injection for chronic discogenic low back pain: preliminary results from a prospective trial. Pain Med. 2016;17:1010‐1022. 26814283 10.1093/pm/pnv053

[152] Akeda K , Ohishi K , Masuda K , et al. Intradiscal injection of autologous platelet‐rich plasma releasate to treat discogenic low back pain: a preliminary clinical trial. Asian Spine J. 2017;11:380‐389. 28670405 10.4184/asj.2017.11.3.380 PMC5481592

[153] Nguyen C , Boutron I , Baron G , et al. Intradiscal glucocorticoid injection for patients with chronic low back pain associated with active discopathy: a randomized trial. Ann Intern Med. 2017;166:547‐556. 28319997 10.7326/M16-1700

[154] Kannan A , Dodwad SN , Hsu WK . Biologics in spine arthrodesis. J Spinal Disord Tech. 2015;28:163‐170. 25978141 10.1097/BSD.0000000000000281

[155] Voss T , Paranjpe AS , Cook TG , Garrison NDW . A short introduction to intellectual property rights. Tech Vasc Interv Radiol. 2017;20:116‐120. 28673648 10.1053/j.tvir.2017.04.007

[156] Sternitzke C . Drug repurposing and the prior art patents of competitors. Drug Discov Today. 2014;19:1841‐1847. 25269415 10.1016/j.drudis.2014.09.016

[157] Garcia AM , Lopez‐Moya JR , Ramos P . Key points in biotechnological patents to be exploited. Recent Pat Biotechnol. 2013;7:84‐97. 23848273 10.2174/1872208311307020002

[158] Santer V . Search patents outside U.S. Biotechnology. 1994;12:552. 7764943 10.1038/nbt0694-552a

